# In Silico Evaluation of a Self-powered Venous Ejector Pump for Fontan Patients

**DOI:** 10.1007/s13239-023-00663-5

**Published:** 2023-03-06

**Authors:** Reza Rasooli, Knut Erik Teigen Giljarhus, Aksel Hiorth, Ingunn Westvik Jolma, Jan Ludvig Vinningland, Charlotte de Lange, Henrik Brun, Henrik Holmstrom

**Affiliations:** 1 Department of Energy Resources, Faculty of Science and Technology, University of Stavanger, 4036 Stavanger, Norway; 2 Department of Mechanical and Structural Engineering and Materials Science, University of Stavanger, 4036 Stavanger, Norway; 3 Department of Chemistry, Bioscience and Environmental Engineering, University of Stavanger, 4036 Stavanger, Norway; 4 Norwegian Research Center (NORCE), Oslo, Norway; 5 Department of Paediatric Radiology, Sahlgrenska University Hospital, Gothenburg, Sweden; 6 Section for Medical Cybernetics and Image Processing, The Intervention Centre, Oslo University Hospital Rikshospitalet, Oslo, Norway; 7 Department of Paediatric Cardiology, Division of Paediatric and Adolescent Medicine, Oslo University Hospital, Oslo, Norway; 8 Institute of Clinical Medicine, Faculty of Medicine, University of Oslo, Oslo, Norway

**Keywords:** Congenital heart disease, Single-ventricle physiology, Fontan hemodynamics, Systemic venous hypertension, Computational fluid dynamics, Fontan venous assist

## Abstract

**Purpose:**

The Fontan circulation carries a dismal prognosis in the long term due to its peculiar physiology and lack of a subpulmonic ventricle. Although it is multifactorial, elevated IVC pressure is accepted to be the primary cause of Fontan's high mortality and morbidity. This study presents a self-powered venous ejector pump (VEP) that can be used to lower the high IVC venous pressure in single-ventricle patients.

**Methods:**

A self-powered venous assist device that exploits the high-energy aortic flow to lower IVC pressure is designed. The proposed design is clinically feasible, simple in structure, and is powered intracorporeally. The device's performance in reducing IVC pressure is assessed by conducting comprehensive computational fluid dynamics simulations in idealized total cavopulmonary connections with different offsets. The device was finally applied to complex 3D reconstructed patient-specific TCPC models to validate its performance.

**Results:**

The assist device provided a significant IVC pressure drop of more than 3.2 mm Hg in both idealized and patient-specific geometries, while maintaining a high systemic oxygen saturation of more than 90%. The simulations revealed no significant caval pressure rise (< 0.1 mm Hg) and sufficient systemic oxygen saturation (> 84%) in the event of device failure, demonstrating its fail-safe feature.

**Conclusions:**

A self-powered venous assist with promising in silico performance in improving Fontan hemodynamics is proposed. Due to its passive nature, the device has the potential to provide palliation for the growing population of patients with failing Fontan.

**Supplementary Information:**

The online version contains supplementary material available at 10.1007/s13239-023-00663-5.

## Introduction

Palliative repair of univentricular heart anomalies necessitates a series of staged open-heart surgical operations aimed at rerouting the systemic venous return directly to the pulmonary arteries, bypassing the single functioning ventricle. The Fontan procedure, first introduced in 1971,^16^ is the final stage of these operations, in which the inferior vena cava (IVC) is anastomosed to the pulmonary arteries via an intra-atrial lateral tunnel or extra-cardiac conduit, resulting in total cavopulmonary connection (TCPC).^11,15,32^ It is estimated that 70 000 patients may be alive worldwide with Fontan circulation today, and this population is expected to double in the next 20 years.^58^ Despite the acceptable early survival rate of the Fontan procedure, the lack of an active subpulmonary ventricle has resulted in an increasing number of patients with failed Fontan physiology^2,30^ and postoperative complications. Fontan-associated liver disease,^12,19^ protein-losing enteropathy,^3^ reduced ventricular filling, and low cardiac output^37,40^ are some of the serious complications encountered after the Fontan procedure. Albeit the physiological associations and causes of these problems are intricate and multifactorial, elevated systemic venous pressure is widely acknowledged to play a paramount role.^53^ The most prevalent theory considers non-pulsatile pulmonary arterial flow as the primary contributing factor to the systemic venous hypertension in Fontan physiology. Lack of pulsatility in pulmonary arterial flow has been shown to cause pulmonary vascular changes in both animal and patient groups,^23,24,33,70^ reduced nitric oxide synthetase and endothelial dysfunction, and finally elevated pulmonary vascular resistance.^36,59^ This highlights the important role of pulmonary flow pulsatility in the Fontan postoperative complications.

The need for new and innovative treatment strategies has repeatedly been highlighted.^34^ Various solutions have been proposed to mitigate the complications associated with high systemic venous pressure (SVP). One of the earliest solutions to the elevated SVP was the fenestration procedure, in which an opening is created between the Fontan conduit and the atrium. A fenestration may improve the early postoperative course by preserving the preload in the systemic circulation and acting as a relief valve for elevated IVC pressure. However, it has no long-term benefits and comes at the price of decreased oxygen saturation and increased risk of systemic embolization and reinterventions.^7^ The main issue with fenestration is its inability to provide driving energy that can be used to assist the cavopulmonary flow. This was the primary motivation for the development of Fontan venous assist devices (FVAD),^10,68^ in which an external energy source is utilized to propel an implanted mechanical pump,^56,63^ similar to conventional ventricle assist devices.

Self-powered Fontan venous assist is an emerging concept that eliminates the need for external energy and thus can provide a passive solution to the Fontan paradox. The primary source of power in this concept is the high-energy arterial flow generated by the native single-ventricle, which can be exploited for venous side assist. Although other alternative energy sources such as skeletal muscle,^35,64^ tissue-engineered contractile conduits,^5^ and atrial cardiomyoplasty^66^ have also been proposed, insufficient energy transfer hinders their implementation in clinical practice for proper venous support. In an attempt to assist a bidirectional Glenn, a Venturi shunt from the systemic brachiocephalic artery to superior vena cava (SVC) was found to be effective in improving the pulmonary flow using computational simulations,^14,65^ but at the expense of elevated SVC pressure, for which modified designs were proposed.^27^ Following the self-powered Fontan palliation strategies, an integrated turbine-pump system was introduced, in which a turbine extracts energy from the aortic flow and transfers it through a common shaft to drive a coupled pump on the venous side.^47^ Despite initial in vitro experiments demonstrating its potential for lowering SVP, the turbine-to-pump leakage for low aortic flow was reported to be significant with a drastic adverse impact on device performance, and therefore remains a great challenge to overcome. On the path toward simpler designs, an injection jet shunt that simply draws flow from the aorta to either the pulmonary arteries or the IVC via a synthetic graft was introduced and demonstrated to provide a reasonable IVC pressure drop using computational simulations.^46,50^ The unsupported aortic graft inside the vessel lumen, on the other hand, renders it clinically impracticable and, more importantly, carries high postoperative risks due to possible significant graft fluctuations. A Venturi shunt from the ascending aorta to the anastomosis of the SVC and pulmonary arteries was also recently proposed to help lower venous pressure.^69^ The computational simulations revealed an insignificant IVC pressure drop of less than 1 mm Hg, as well as a highly unbalanced pulmonary flow distribution.

Most FVADs are powered extracorporeally, which severely limits their long-term usability due to complications connected to driveline infection. More importantly, the previously proposed self-powered venous assists also either lack clinical feasibility or pose a high postoperative risk due to their complex structures. To address these, we propose a clinically practicable Fontan venous assist that requires no external source of energy to function and contains no rotating machinery, thus having the potential to palliate the detrimental effects of Fontan circulation.

**Figure 1:**
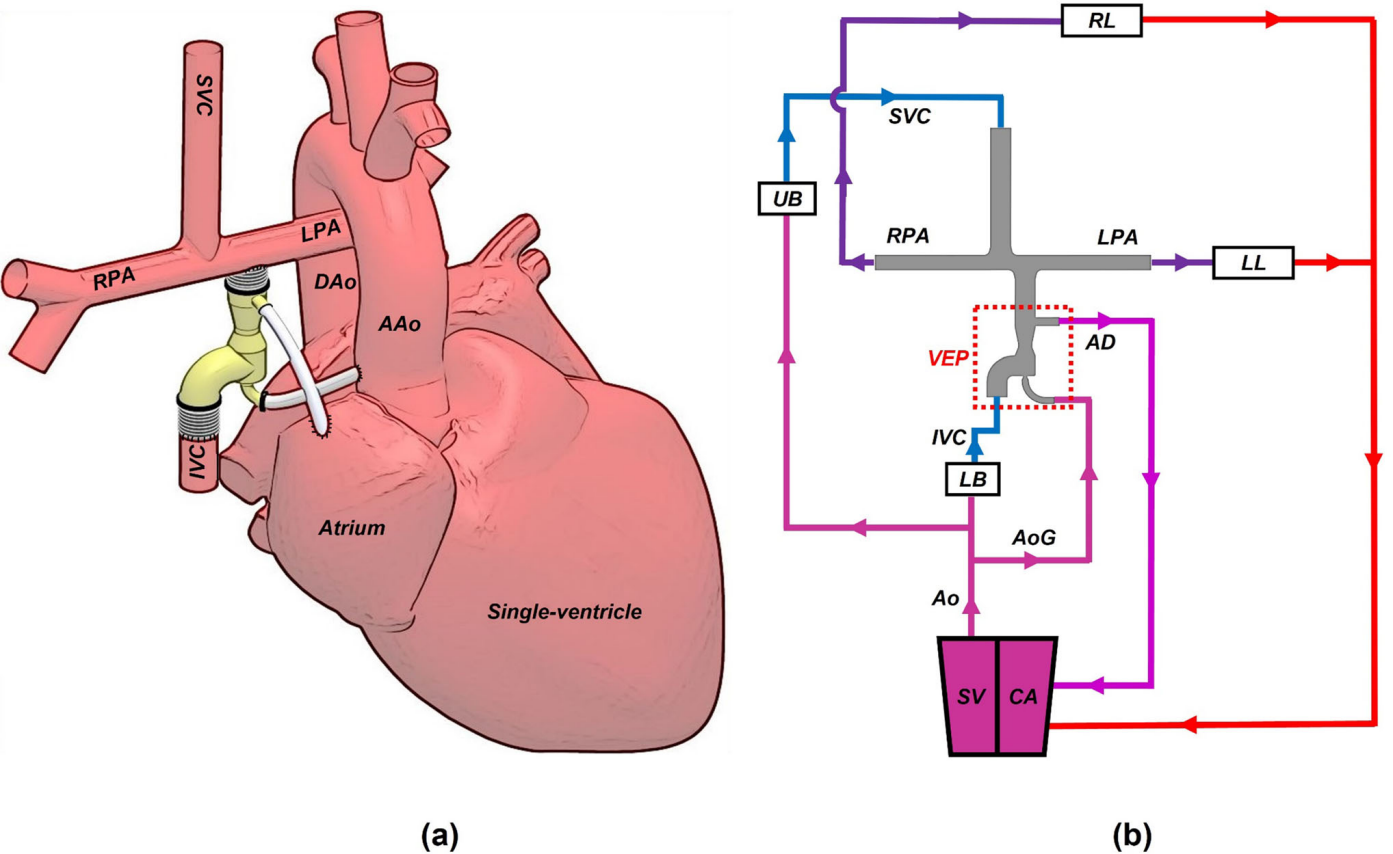
Schematic anatomical illustration (a) and circulation loop (b) of the assisted Fontan using the proposed self-powered venous ejector pump (VEP). The VEP device is powered intracorporeally and contains no rotating machinery. The VEP utilizes the synergistic effect of high-energy aortic flow and low atrial pressure to provide a significant IVC pressure drop as an effective palliation for Fontan patients with systemic venous hypertension. *IVC* Inferior vena cava, *SVC* superior vena cava, *RPA* right pulmonary artery, *LPA* left pulmonary artery, *AAo* ascending aorta, *DAo* descending aorta, *UB* upper body, *LB* lower body, *RL* right lung, *LL* left lung, *AoG* aortic graft, *AD* atrial discharge, *SV* single-ventricle and, *CA* common atrium. The line colors on the right side schematically represent the blood oxygen saturation with blue and red being the systemic venous and pulmonary veins oxygen levels, respectively.

Our proposed venous ejector pump (VEP) utilizes the high-energy aortic flow powered by the functioning single-ventricle to generate a suction effect for the IVC blood flow (Fig. 1), akin to popular industrial ejector pumps used in oil and gas recovery,^18^ and therefore can: (1) improve the cavopulmonary flow pulsatility to mitigate the complications associated with the non-pulsatile pulmonary flow; (2) increase the pulmonary flow without elevating the vena caval pressure; (3) provide a significant drop in the IVC pressure (pressure difference between before and after including the VEP) and maintain a chronically low venous pressure to alleviate the high SVP problems; (4) increases preload in the systemic circulation and (5) be readily fabricated or 3D printed in any research laboratory or clinical center at low costs.

Computational fluid dynamics (CFD) is employed in this study to assess the performance of the VEP in reducing the IVC pressure and improving the pulmonary flow in both idealized and patient-specific TCPC models using clinically relevant flow and pressure conditions. The impact of key geometrical parameters was assessed based on the IVC and SVC pressure, aortic graft flow rate, and the systemic arterial oxygen saturation using the one-diameter inlets offset idealized TCPC model. The best performing candidate were then applied to the patient-specific models as well as idealized TCPCs with zero and 0.5 inlet offsets. The performance was assessed during both full assist and failure of device situations.

## Methods

### Venous Ejector Pump (VEP)

**Figure 2:**
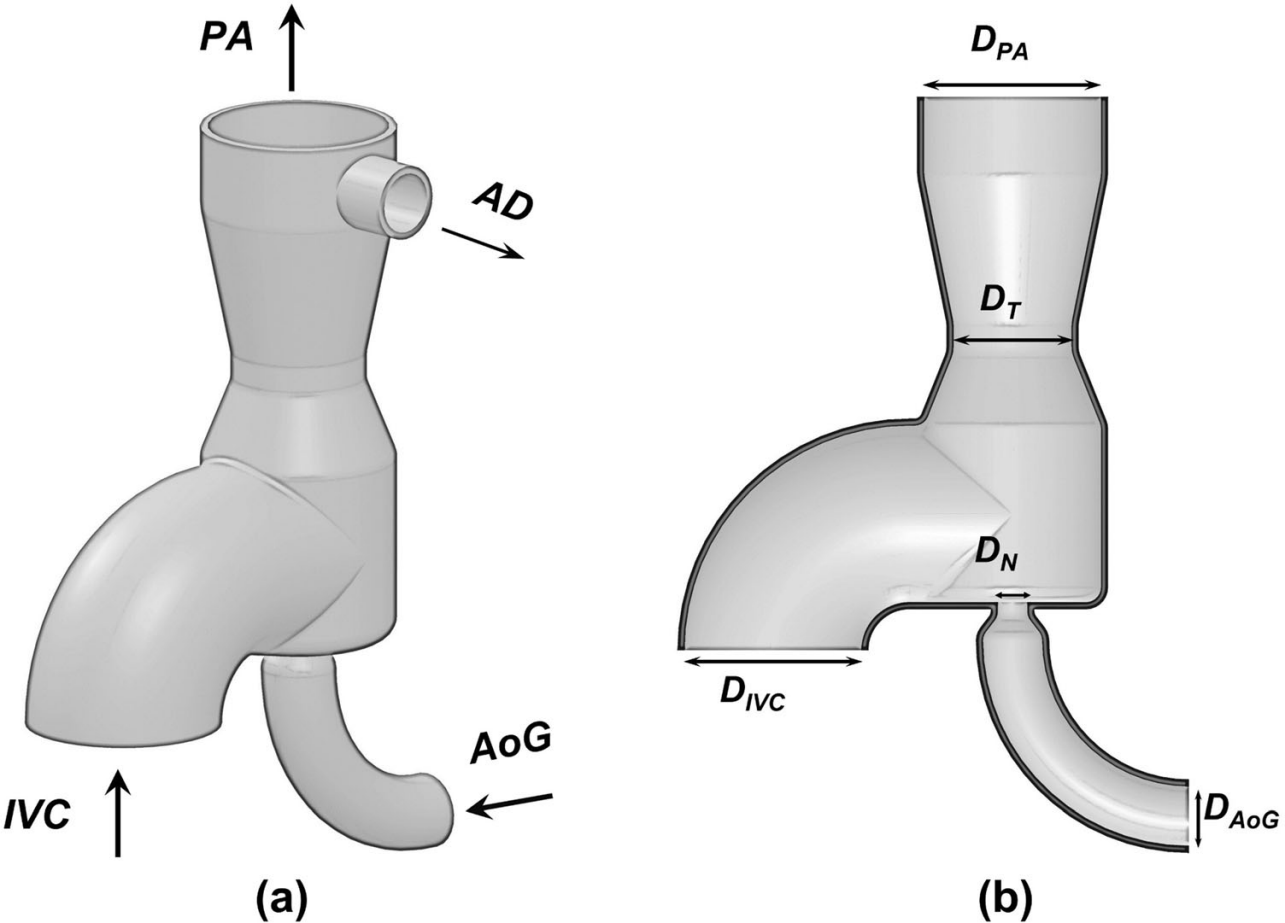
3-dimensional geometry of the proposed self-powered venous ejector pump. *IVC* Inferior vena cava, *SVC* superior vena cava, *RPA* right pulmonary artery, *LPA* left pulmonary artery, *AoG* aortic graft, *AD* atrial discharge, *PA* pulmonary artery, *D*_*IVC*_ IVC inlet diameter, *D*_*AoG*_ aortic graft inlet diameter, *D*_*N*_ aortic graft nozzle diameter, *D*_*T*_ throat diameter, and *D*_*PA*_ pulmonary artery outlet diameter.

The proposed venous ejector pump has two inlets for collecting IVC blood and connecting the aortic graft, as well as two outlets for venous blood discharge into the pulmonary arteries and the atrium (Fig. 2). The device employs two important mechanisms to lower the IVC pressure: the Venturi effect from the high-velocity aortic graft jet and the atrium discharge flow suction. The aortic graft (AoG) is a left-to-right shunt akin to aortopulmonary shunts that is anastomosed to the ascending aorta and injects a fraction of cardiac output into the low-energy IVC flow connected to the IVC port of the VEP. The atrial discharge (AD) acts as a right-to-left shunt similar to fenestration which drains a fraction of the systemic venous return to the atrium (Fig. 1). The atrial discharge provides four major advantages: 1) it increases preload in the systemic circulation 2) provides additional IVC pressure drop (pressure difference between pre and post VEP inclusion); 3) it prevents elevated SVC pressure resulting from IVC flow boosting; 4) it maintains pre-operative IVC and SVC pressures in the event of device failure due to aortic graft occlusion. The IVC inlet and pulmonary outlet ports are 12 mm in diameter, corresponding to the typical idealized size for the pediatric population. The aortic flow port has an inlet diameter of 4 mm, which is within the acceptable range for the systemic-to-pulmonary shunts and thus has clinical feasibility.^46^

### Idealized and Patient-Specific Total Cavopulmonary Connection Models

**Figure 3:**
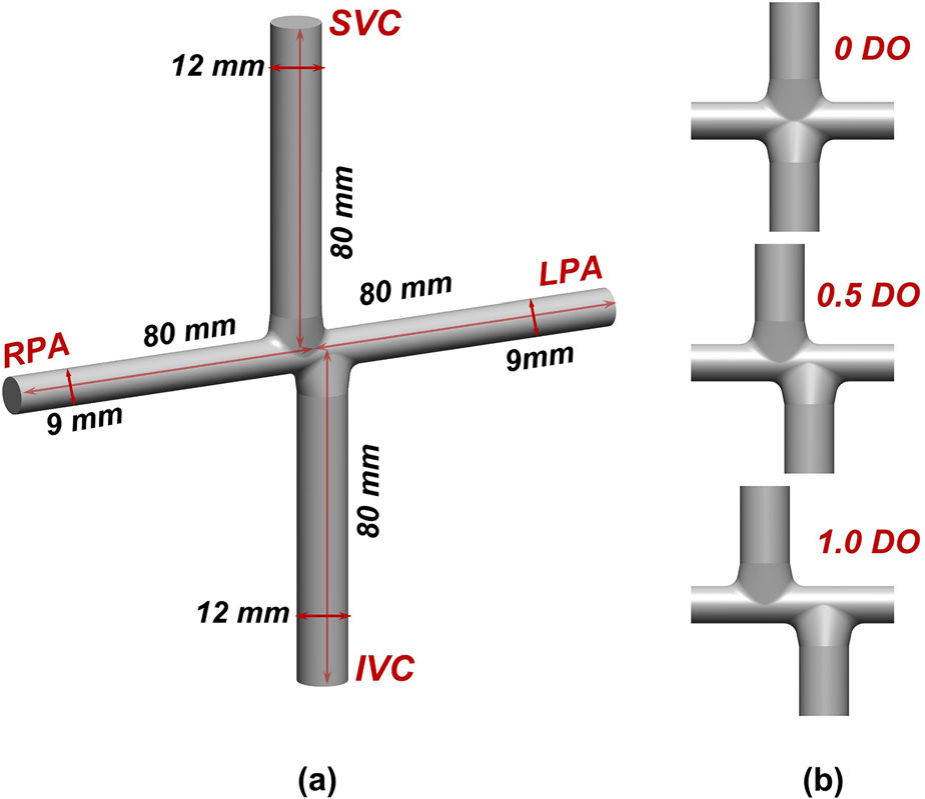
3-Dimensional geometries of idealized total cavopulmonary connections (TCPC). (a) represents the dimensions and (b) depicts the impingement zone for zero, half, and one diameter offset. *IVC* Inferior vena cava, *SVC* superior vena cava, *RPA* right pulmonary artery, *LPA* left pulmonary artery, *DO* diameter offset.

**Figure 4:**
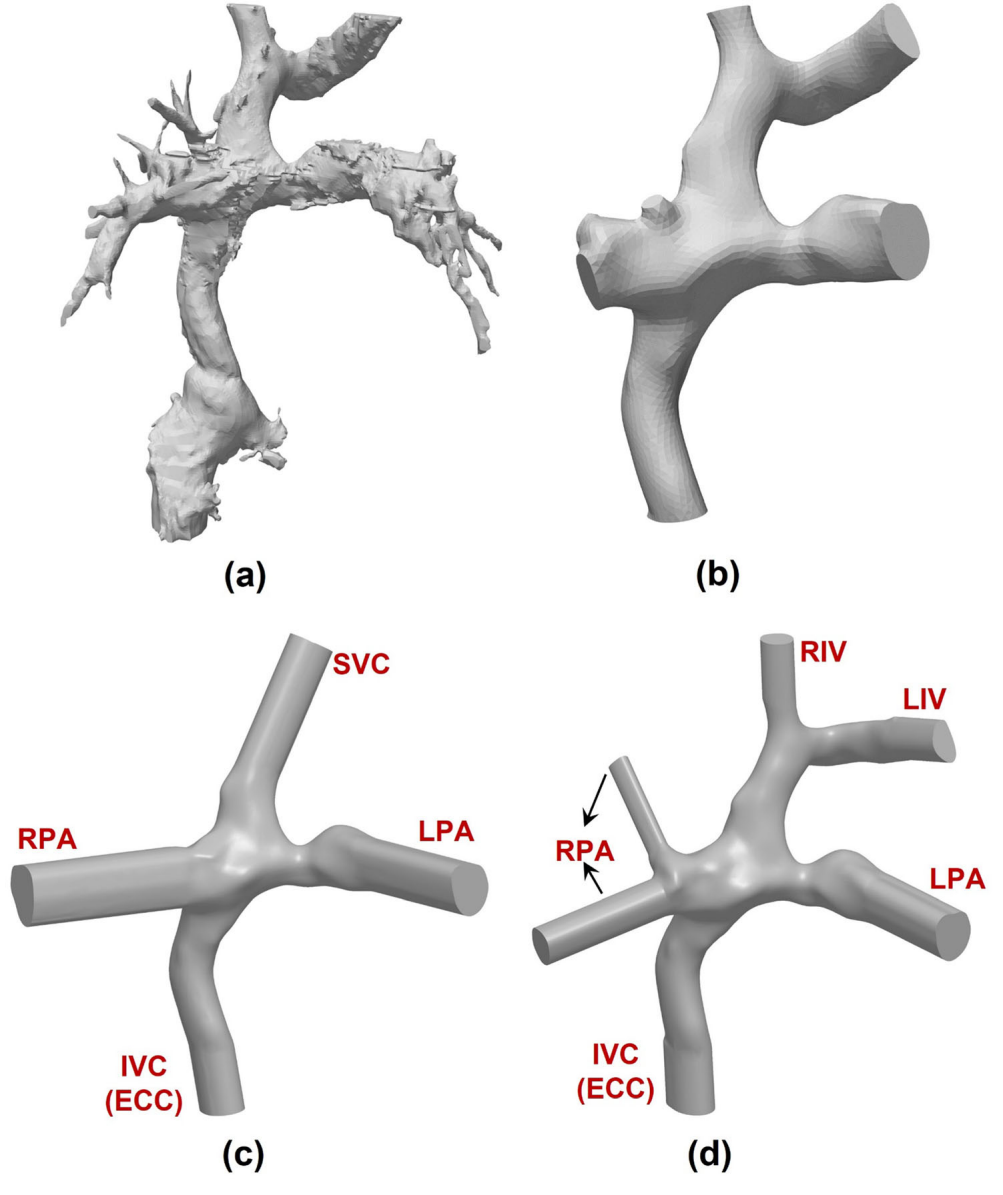
Anatomical representation of the 3D reconstructed patient-specific total cavopulmonary connection. (a) raw 3D reconstructed extracardiac Fontan patient-specific geometry, (b) smoothed 3D model, (c) simplified patient-specific TCPC model (PSC1), and (d) complex patient-specific TCPC model (PSC2). *IVC* Inferior vena cava, *SVC* superior vena cava, *RPA* right pulmonary artery, *LPA* left pulmonary artery, *ECC* extra-cardiac conduit, *RIV* right innominate vein, and *LIV* left innominate vein.

Three intersecting double-inlet double-outlet geometries with zero, 0.5, and one diameter offset (0 DO, 0.5 DO, and 1 DO) and non-identical inlet/outlet circular cross-sections representing idealized TCPCs were designed using Autodesk inventor (Autodesk, California, USA) for computational simulations (Fig. 3). The inlets (SVC & IVC) and outlets (RPA & LPA) diameters were considered to be 12 mm and 9 mm, respectively. The vena cavae and pulmonary artery anastomosis intersections were rounded with 3-mm fillets. Sufficient extensions were considered for all inlets/outlets to minimize the effects of boundary conditions uncertainties on the impinging zone and improve the solver stability.^39^ For the patient-specific TCPC model, a clinical magnetic resonance (MR) exam (1.5 T MR, Siemens Aera, Siemens Erlangen, Germany) of a 16-year-old male patient with extra-cardiac Fontan conduit was used (Fig. 4a and Fig. 4b). A respiratory and ECG navigated 3D whole heart balanced steady-state free precession sequence was performed in a coronal plane covering the heart and thorax. Standard clinical MRI DICOM images were segmented and modeled with Mimics and 3-Matic software (Materialise, Belgium) applying thresholding with manual corrections as appropriate according to the images. The final geometrical smoothing was performed using the free software meshmixer (Autodesk Research, USA). Two geometries with different complexity levels were generated for the patient-specific TCPC model. Patient-specific case 1 (PSC1, Fig. 4c) represents a simplified version of the patient model while patient-specific case 2 (PSC2, Fig. 4d) provides more geometrical details including the right pulmonary artery branching and innominate veins.

### Geometrical Design Parameters

To assess the ideal operating range and performance capabilities of the VEP, three key geometrical design parameters are identified: 1) aortic nozzle diameter; 2) throat size, and 3) atrial discharge diameter (Fig. 2). Following the design of experiments, reasonable values are considered for each parameter as stated below:

- Nozzle diameter: 1.5 mm, 2 mm, 2.5 mm, 3 mm
- Throat diameter: 6 mm, 8 mm, 10 mm, 12 mm
- Atrial discharge diameter: 4 mm, 5 mm, 6 mm

The atrial discharge diameter values were decided based on typical fenestration sizes used in Fontan patients.^28^ The throat diameter values correspond to a variation of half to full IVC diameter. Throat sizes smaller than half the IVC diameter would place a high resistance on the systemic venous flow and thus are not considered. The aortic graft inlet size was considered to be in line with the common clinical practice for aortopulmonary shunts.^49^ The aortic nozzle sizes were selected based on the earlier studies in the literature.^46,50^ Due to the presence of a left-to-right shunt in the VEP (aortic graft), an extra load is placed on the single-ventricle as a result of reduced systemic flow. These shunts result in a pulmonary (Qp) to systemic flow (Qs) ratio of higher than one (Qp/Qs > 1). Left-to-right shunts with Qp/Qs > 1.5 lead to development of ventricular dysfunction, atrial enlargement, and left ventricular volume overload.^13,52^ Assuming the aortic graft as the only shunt in the device, the pulmonary flow can be written as:

1 $$Q_{{\text{P}}} \; = \;Q_{{\text{s}}} \; + \;Q_{{{\text{shunt}}}}$$

Calling the physiological limit for the pulmonary to systemic flow ratio:

2 $$\frac{{Q_{{\text{P}}} }}{{Q_{{\text{S}}} }} < 1.5 \to \frac{{Q_{{\text{s}}} + Q_{{{\text{shunt}}}} }}{{Q_{{\text{S}}} }} <1.5\; \to \;1\; + \;\frac{{Q_{{{\text{shunt}}}} }}{{Q_{{\text{S}}} }}<1.5\;\mathop{\longrightarrow}\limits^{{{\text{yields}}}}\;\frac{{Q_{{{\text{shunt}}}} }}{{Q_{{\text{S}}} }} < 0.5$$

This indicates that the flow inside the left-to-right shunt (aortic graft) must be lower than half the systemic flow (aortic graft flow criterion) to avoid the development of aforementioned complications. Since the aortic graft flow is a strong function of nozzle diameter, we assumed that the effect of the throat and atrial discharge diameter on the aortic graft flow is negligible. Thus, the impact of nozzle size is evaluated separately with fixed throat and atrial discharge diameters of 8 mm and 4 mm, respectively, which resulted in a total of 15 combinations of geometries. The 1DO idealized TCPC model was used to determine the most optimal geometrical settings which were then applied to the patient-specific and idealized with different inlet offset TCPC models. The most optimal setting was considered the case which satisfies the following criteria:

- Aortic graft flow criterion (*Q*_AoG_/*Q*_S_ < 0.5)
- Systemic arterial oxygen concentration higher than 80% during device full assist
- Systemic arterial oxygen concentration higher than 80% in the event of aortic graft occlusion
- Preserving pre-VEP TCPC baseline conditions in the event of device full failure (occlusion of both aortic graft and atrial discharge)

### Computational Fluid Dynamics (CFD)

The steady-state simulations were conducted using the open-source CFD simulation software OpenFOAM, version 2112.^26,67^ Reynolds-averaged Navier Stokes (RANS) equations incorporating the k-ω shear stress transport (SST) turbulence model^42^ were utilized to account for the turbulent flow characteristics present at the high-velocity jet flow exhausting from the aortic graft nozzle (Re ~ 2600 for the aortic nozzle diameter of 2.5 mm), which has been reported to provide the best agreement with the theoretical solution for circular free jets.^31^ The SIMPLE algorithm was used for pressure–velocity coupling, with second-order discretization schemes applied for spatial discretization. The iterations were continued until the scaled residual reductions of less than 10^–4^ in velocity, pressure, and turbulence quantities are achieved. In addition to the residual reduction, integral quantities such as mass flow rate were also monitored to ensure convergence. The computational mesh consisted of primarily unstructured hexahedral cells generated using snappyHexMesh, with a cell size of approximately 0.1 mm. This gave a maximum and average non-dimensional distance to the wall of $y_{\max }^{ + } < 2$ and $y_{{{\text{avg}}}}^{ + } < 0.3$. Other grid parameters were also of suitable quality, with non-orthogonality less than 65, skewness less than 4 and maximum aspect ratio less than 8. The mesh is additionally refined near the walls using five prismatic cell layers with an expansion ratio of 1.15. A mesh independence study using three grid resolutions of coarse (1.7 M cells), medium (3.3 M cells) and, fine (6.2 M cells) was conducted to ensure that the final computational results are free of discretization error. The grid convergence index (GCI)^54^ for quantities of interest such as IVC pressure and pulmonary flow was monitored for each case to ensure that the solutions are within the asymptotic range of convergence. The medium grid resolution was selected for the simulations. Blood was modeled as a Newtonian fluid with a constant density and dynamic viscosity of 1060 kg/m^3^ and 0.0035 Pa.s, respectively.

### Boundary Conditions

A fully developed velocity profile was assigned for all the inlets of the idealized TCPC models. The total vena caval blood flow rate in both idealized and patient-specific TCPC models was considered to be 2.1 L/min, which is an acceptable estimate for the pediatric population.^57^ A 60/40 flow split between IVC and SVC in the idealized and PSC1 models was considered to simulate the postoperative pediatric Fontan stage. In the PSC2 model, a 20% flow split was assigned for each right and left innominate vein. Static pressure of 65 mm Hg was assigned at the inlet of the aortic graft to represent the mean arterial pressure.^69^ Although the aortic pressure is strongly pulsatile, the device performance is strongly correlated with the time-averaged flow characteristics. The validity of using a time-averaged pressure for the aortic graft inlet was investigated. Please refer to electronic supplementary material for more details. A uniform velocity profile was considered for the aortic graft inlet to best describe the velocity profile shape at the aortic graft and aorta anastomosis since it acts as a starting flow. Constant outlet pressures of 10 mm Hg with zero gradient velocity were considered for the right and left pulmonary arteries to generate a hypertensive venous condition. The atrium discharge outlet pressure was set to 5 mm Hg to represent the typical time-averaged Fontan atrial pressure in the pediatric group.^45^ All walls were assumed to be rigid with no-slip velocity conditions. The failure of device due to complete aortic graft occlusion was simulated by assigning zero flow at the aortic graft inlet.

### Oxygen Saturation Monitoring

**Figure 5:**
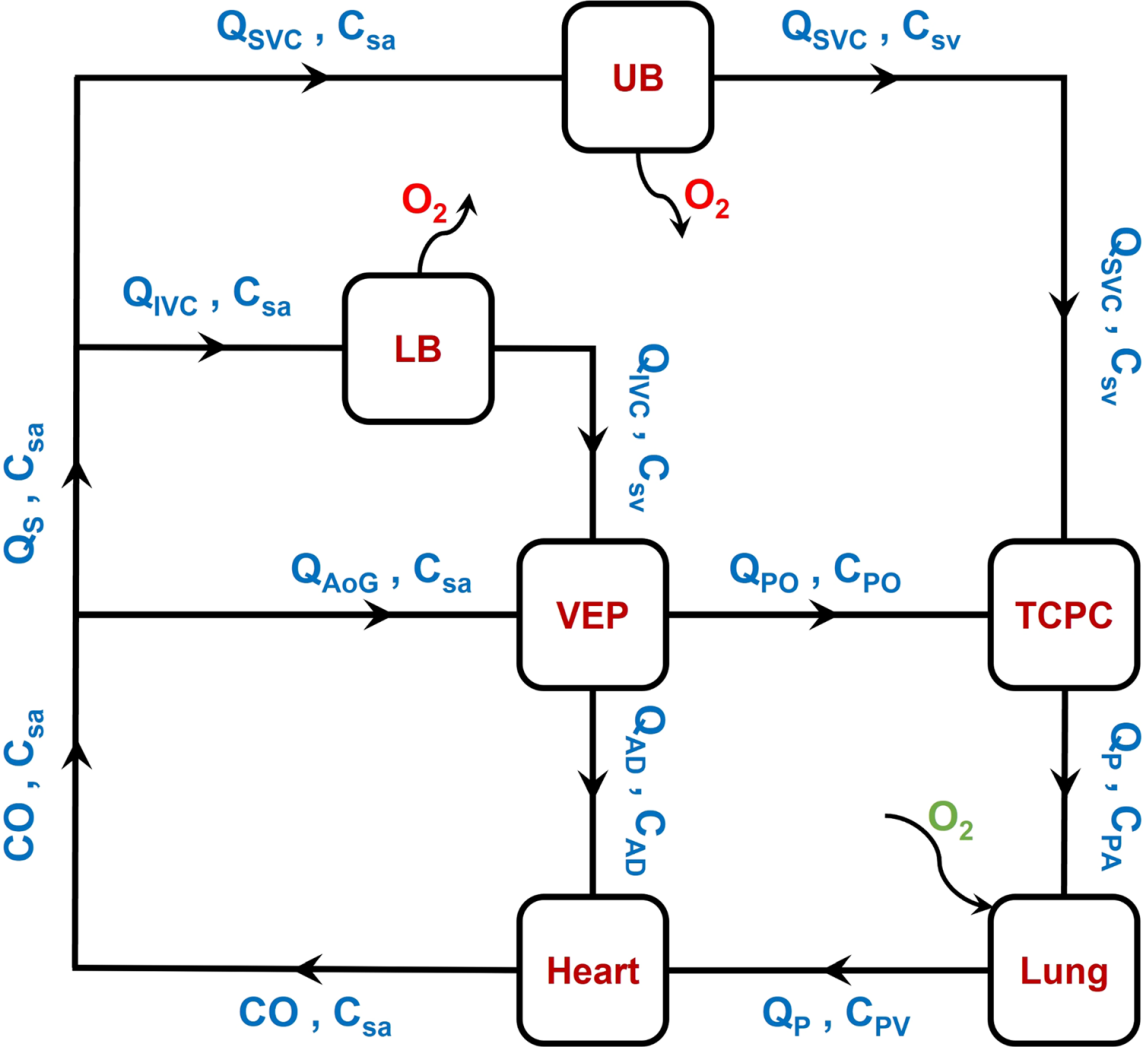
Oxygen transport diagram in a VEP-assisted Fontan circulation. *Q* flow rate, *C* oxygen concentration, *LB* lower body, *UB* upper body, *VEP* venous ejector pump, *TCPC* total cavopulmonary connection, *CO* cardiac output, *C*_*sa*_ systemic arterial oxygen concentration, *Q*_*S*_ systemic flow rate, *IVC* inferior vena cava, *SVC* superior vena cava, *C*_*SV*_ systemic venous oxygen saturation, *AoG* aortic graft, *AD* atrial discharge, *PO* pump outlet, *Q*_*P*_ Pulmonary flow, *PA* pulmonary arteries, *PV* pulmonary veins.

The presence of an atrial discharge in the device causes slight systemic arterial oxygen desaturation which has to be tracked. Figure 5 represents the diagram of oxygen transport in a VEP-assisted Fontan circulation. Considering Fig. 5, the oxygen mass conservation for the heart can be written as:

3 $$Q_{{\text{P}}} C_{{{\text{PV}}}} \; + \;Q_{{{\text{AD}}}} C_{{{\text{AD}}}} \; = \;{\text{CO}} \cdot C_{{{\text{sa}}}}$$

where *Q*_P_, *Q*_AD_, *C*_PV_, *C*_AD_, *C*_sa_ and, CO represent the pulmonary flow rate, atrial discharge flow rate, pulmonary veins oxygen concentration, oxygen concentration in the atrial discharge graft, systemic arterial oxygen concentration, and cardiac output, respectively. The oxygen concentration inside the atrial discharge can be defined as:

4 $$C_{{{\text{AD}}}} \; = \;C_{{{\text{sv}}}} \; + \;m\left( {C_{{{\text{sa}}}} \; - \;C_{{{\text{sv}}}} } \right)$$

where *C*_sv_ is the systemic venous oxygen concentration. Coefficient m is a dimensionless variable that indicates the arterial oxygen contribution to the atrial discharge. *A m* = 0 states no arterial oxygen contribution to the atrial discharge (*C*_AD_ = *C*_sv_) and m = 1 indicates that the whole atrial discharge flow is from the aortic graft (*C*_AD_ = *C*_sa_). To determine the value of m, the passive scalar transport equation was computationally solved for each case. A concentration of 1 at the aortic graft inlet and zero elsewhere was considered as the inlet boundary conditions. The walls were assumed to be impermeable with zero flux boundary condition. The surface-averaged concentration at the atrial discharge was considered as the value of coefficient *m*. The relation between systemic venous and arterial oxygen concentrations for the Fontan patients is suggested in the literature as^1,25^:

5 $$C_{{{\text{sv}}}} \; = \;0.6 \cdot C_{{{\text{sa}}}}$$

Considering Eqs. 3–5, the systemic arterial oxygen concentration can be written as:

6 $$C_{{{\text{sa}}}} \; = \;\frac{{Q_{{\text{P}}} \cdot C_{{{\text{PV}}}} }}{{{\text{CO}}\; - \;\left( {0.6\; + \;0.4\;{\text{m}}} \right)Q_{{{\text{AD}}}} }}$$

All flow rate values are taken from the converged CFD simulations. The value for the pulmonary vein oxygen concentration (*C*_PV_) was considered to be 95% to avoid overestimation.

## Results

### CFD Solver Validation

To increase confidence in our computational simulations, the CFD solver was validated against device-specific experimental data conducted in our laboratory as well as the FDA nozzle benchmark test.^22^ The details of the validations are provided in the electronic supplementary material for brevity.

### Impact of Aortic Nozzle Size

**Figure 6:**
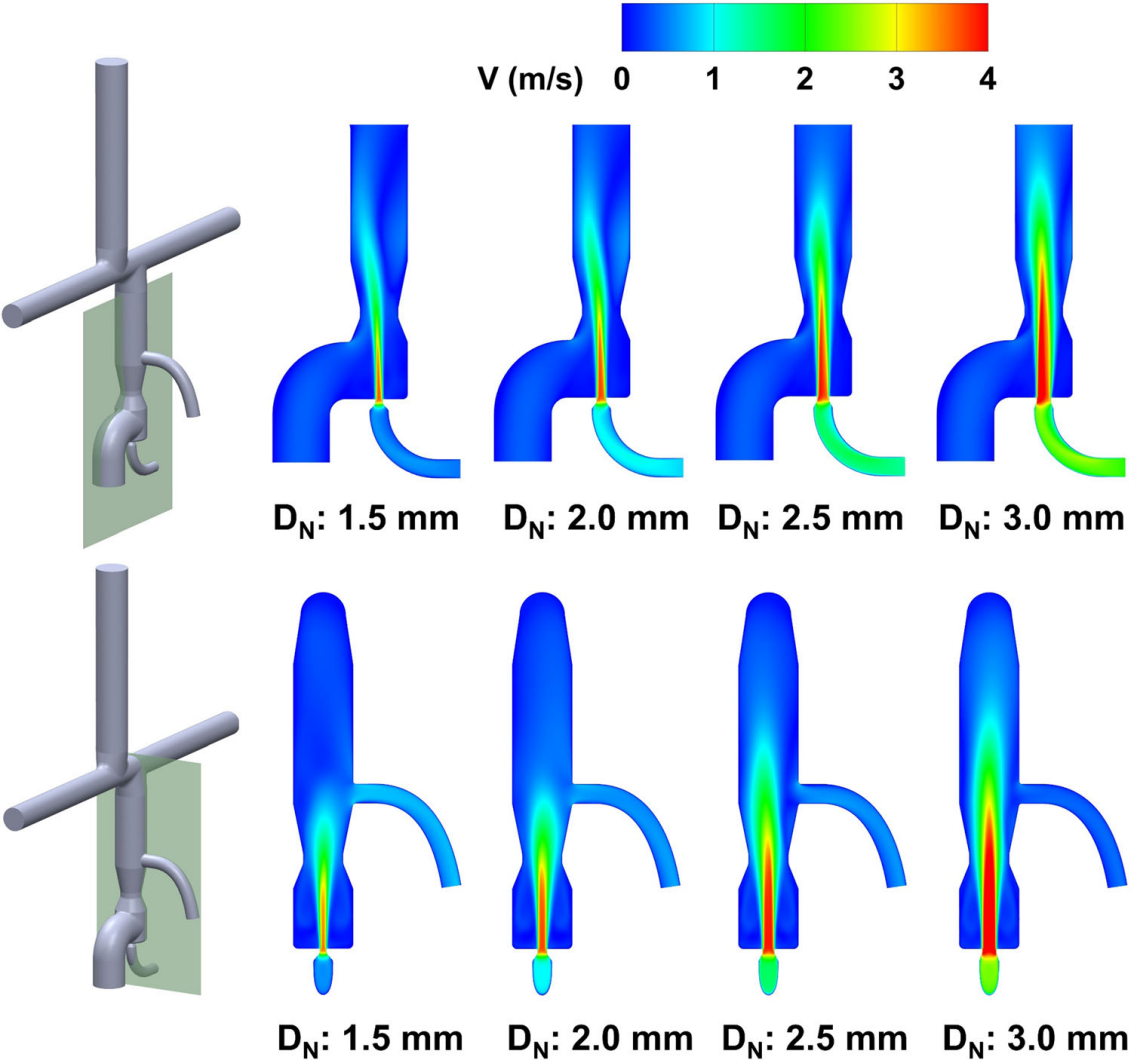
Velocity profile contours for different aortic nozzle diameters at two cross-sections. The top and bottom cross-sections are congruent with the symmetric plane of the aortic graft and the atrial discharge, respectively. The throat and the atrial discharge sizes are 8 mm and 4 mm, respectively, in these simulations. *V* velocity magnitude, *D*_*N*_ aortic nozzle diameter.

**Figure 7:**
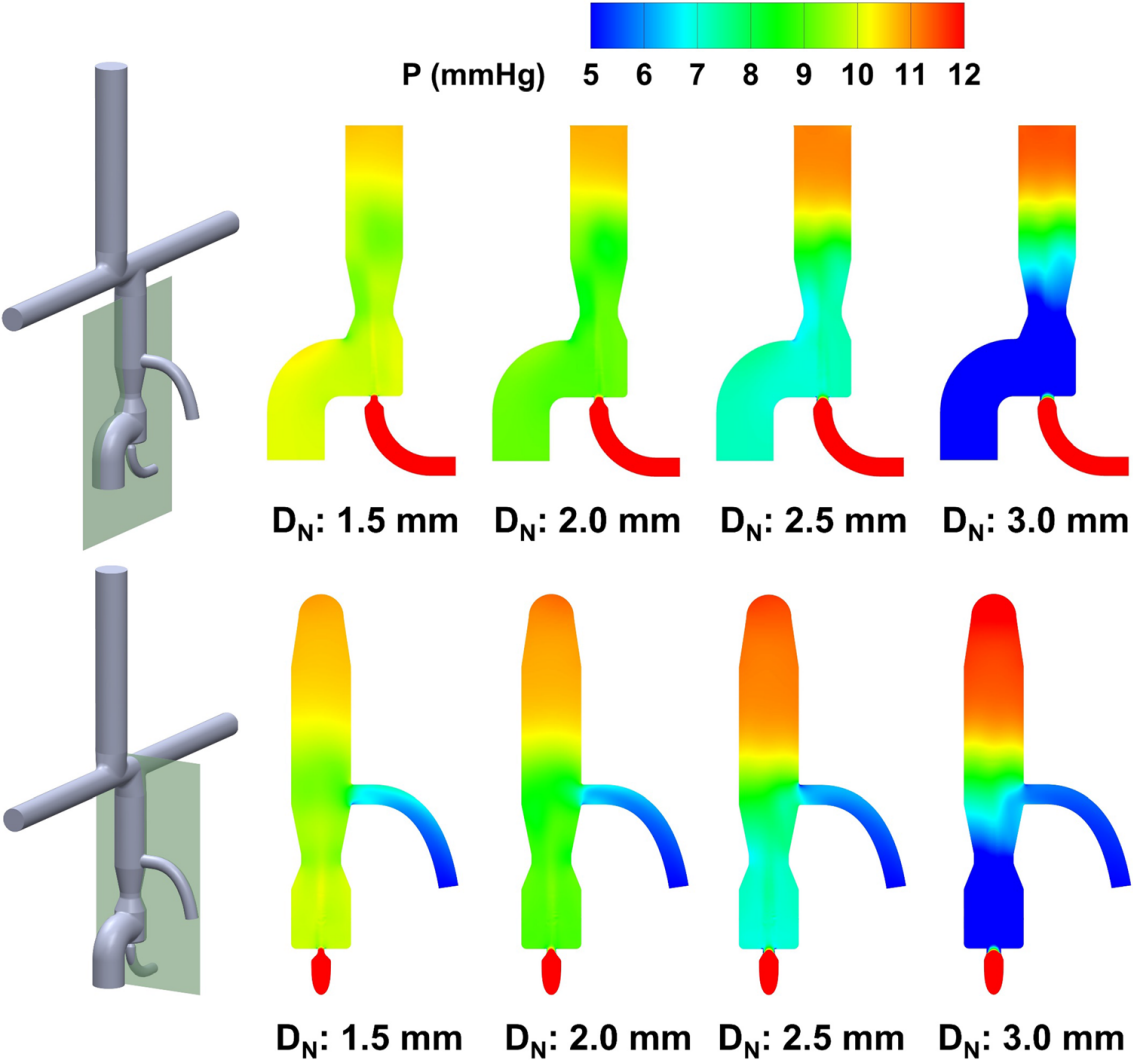
Pressure profile contours for different aortic nozzle diameters at two cross-sections. The top and bottom cross-sections are congruent with the symmetric plane of the aortic graft and the atrial discharge, respectively. The throat and the atrial discharge sizes were 8 mm and 4 mm, respectively, in these simulations. *P* pressure, *D*_*N*_ aortic nozzle diameter.

**Figure 8:**
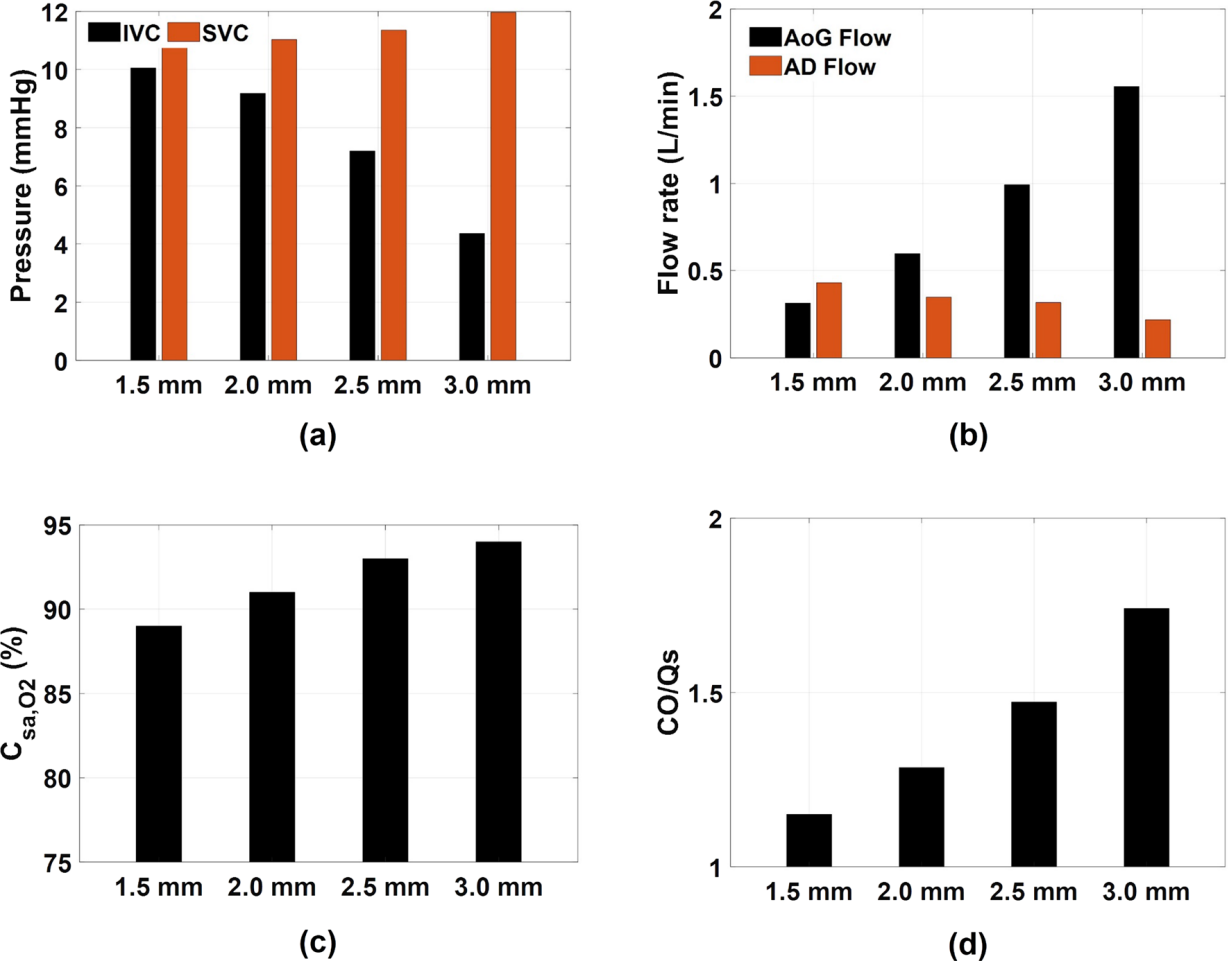
Impact of aortic nozzle diameter on the IVC and SVC pressure (a), aortic graft and atrial discharge flow rate (b), systemic arterial oxygen saturation (c), and the ratio of cardiac output over the systemic flow rate (d). *dP* pressure change as compared to baseline (no VEP), *IVC* inferior vena cava, *SVC* superior vena cava, *AoG* aortic graft, *AD* atrial discharge, *C*_*sa, O2*_ systemic arterial oxygen saturation, *CO* cardiac output, and *Q*_*S*_ systemic flow rate.

The performance of VEP with different nozzle diameters is evaluated using the 1DO idealized TCPC model by monitoring the IVC and SVC pressure, systemic oxygen saturation, and aortic graft flow criterion. The baseline pressure values (TCPC circulation without VEP) for all the TCPC models used in this study are presented in Table 1. Figures 6 and 7 show the impact of different aortic nozzle diameters of 1.5 mm, 2 mm, 2.5 mm, and 3 mm on the velocity and pressure field, respectively. To plot the contours, two cross-sections congruent with the symmetric planes of the aortic graft (top row in Fig. 6 and Fig. 7) and the atrial discharge (bottom row in Fig. 6 and Fig. 7) were considered. In these simulations, the throat and atrial discharge sizes are 8 mm and 4 mm, respectively. The summary of key hemodynamic parameters is provided in Fig. 8 for different nozzle sizes. Larger aortic nozzles produced higher jet flow, resulting in a greater suction effect and consequently lower IVC pressure. Doubling the aortic nozzle diameter (1.5 to 3.0 mm) resulted in an almost fivefold increase in the aortic graft flow rate (0.315 to 1.555 L/min, see Fig. 8) and more than eightfold IVC pressure drop (0.8 to 6.5 mm Hg, see Fig. 8). Even though the 3 mm nozzle outperformed other cases in terms of IVC pressure drop (6.5 mm Hg), the ratio of cardiac output (CO) to systemic flow (Q_S_) was 1.741, which is significantly higher than the acceptable value of 1.5. Such high aortic steals exert a significant load on the single functional ventricle, which can result in ventricular remodeling, diminished systolic function and ejection fraction, and, eventually, heart failure. This ratio was lower than 1.5 for other nozzle diameters which highlights their clinical feasibility. Interestingly, an inverse relationship was observed between the atrial discharge flow and the aortic nozzle size, in which lower atrial discharge flow rates were associated with larger nozzle diameters. This can be justified by the fact that larger nozzles produce higher flow rates, which causes higher velocity at the throat and, consequently, lower pressure. This leads to a smaller pressure gradient between the VEP and the atrium, as also evident in Fig. 7, and thus lower flow rates. More importantly, higher aortic steals were accompanied by elevated SVC pressure, owing to the impingement of IVC and SVC flows. The 1.5 mm nozzle was the only case with a decreased SVC pressure due to the low aortic steal which was smaller than the atrial discharge flow. This underscores the important role of atrial discharge in preventing a sharp rise in the SVC pressure during venous assist with a boosted IVC blood flow.

**Table 1:** Venous pressure values for the baseline condition (no VEP) for all TCPC models used in this study.

	0-DO	0.5-DO	1-DO	PSC1	PSC2
*P*_IVC_ (mm Hg)	11.006	10.825	10.850	10.187	10.284
*P*_SVC_ (mm Hg)	11.228	10.999	10.868	10.185	10.355

0-DO, 0.5-DO, and 1-DO represent zero, half and one diameter offset idealized TCPC models, respectively. PSC1 and PSC2 represent patient-specific case 1 and 2, respectively. IVC: inferior vena cava, SVC: superior vena cava

**Figure 9:**
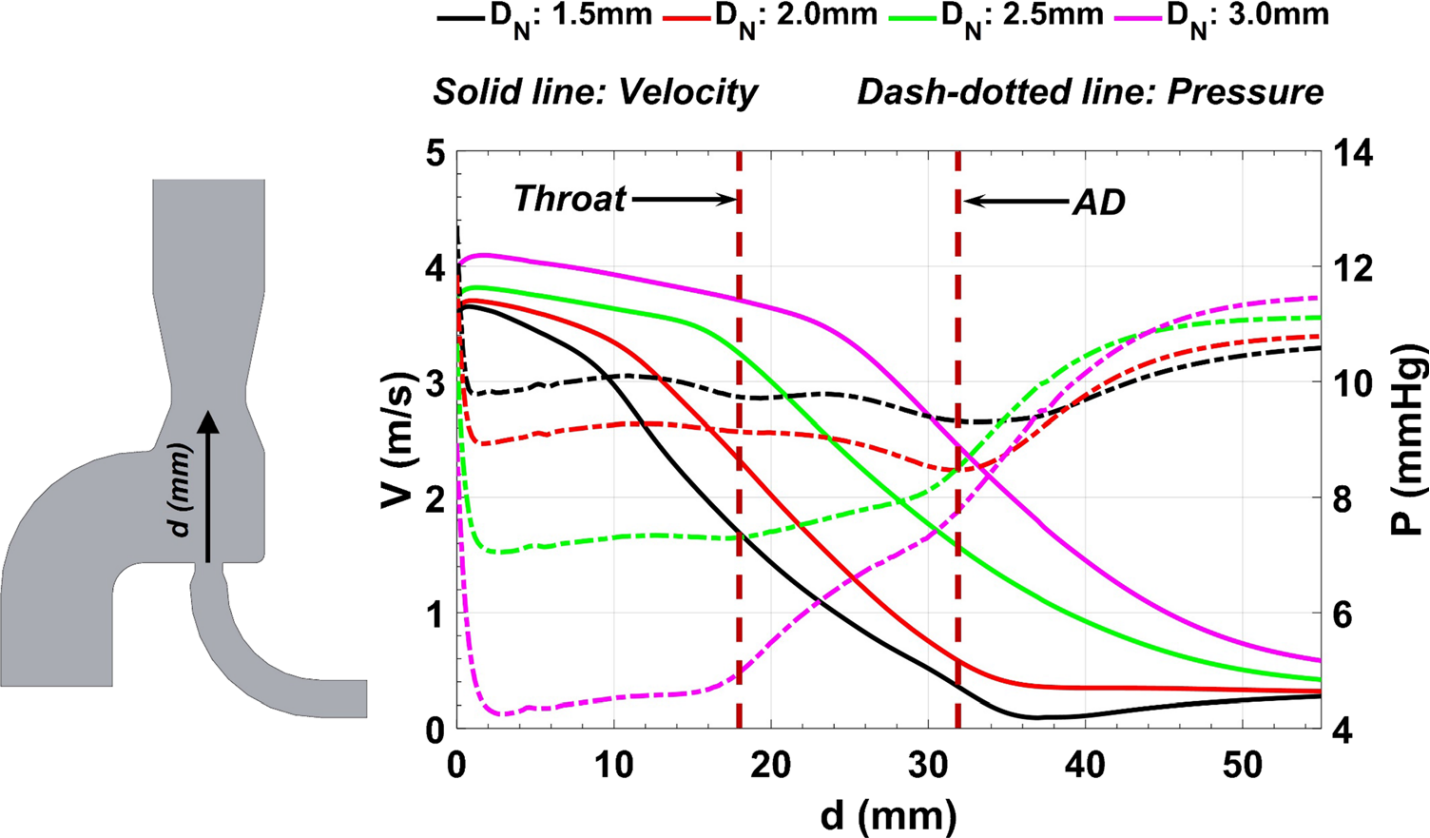
Centerline velocity and pressure variations for four different nozzle diameters. The throat and atrial discharge diameters are 8 mm and 4 mm, respectively. *d* Centerline distance from the tip of the aortic nozzle, *V* velocity magnitude, *P* pressure, *D*_*N*_ aortic nozzle diameter, *AD* atrial discharge.

The velocity and pressure variations on the aortic jet centerline are presented in Fig. 9. The maximum jet velocity rises slightly as the nozzle becomes larger, resulting in a more turbulent jet flow profile, a greater breakdown length, and a lower expansion rate. For the pressure profile, three areas can be identified: an area in the vicinity of the aortic jet tip with a sharp pressure drop, followed by a second area with nearly constant pressure, and a final area with rising pressure. Larger pressure variations are observed as the nozzle enlarges, contributing to more complex hemodynamics inside the device. The details of the simulation results are provided in the electronic supplementary material.

### Impact of the Throat and Atrial Discharge Size

**Figure 10:**
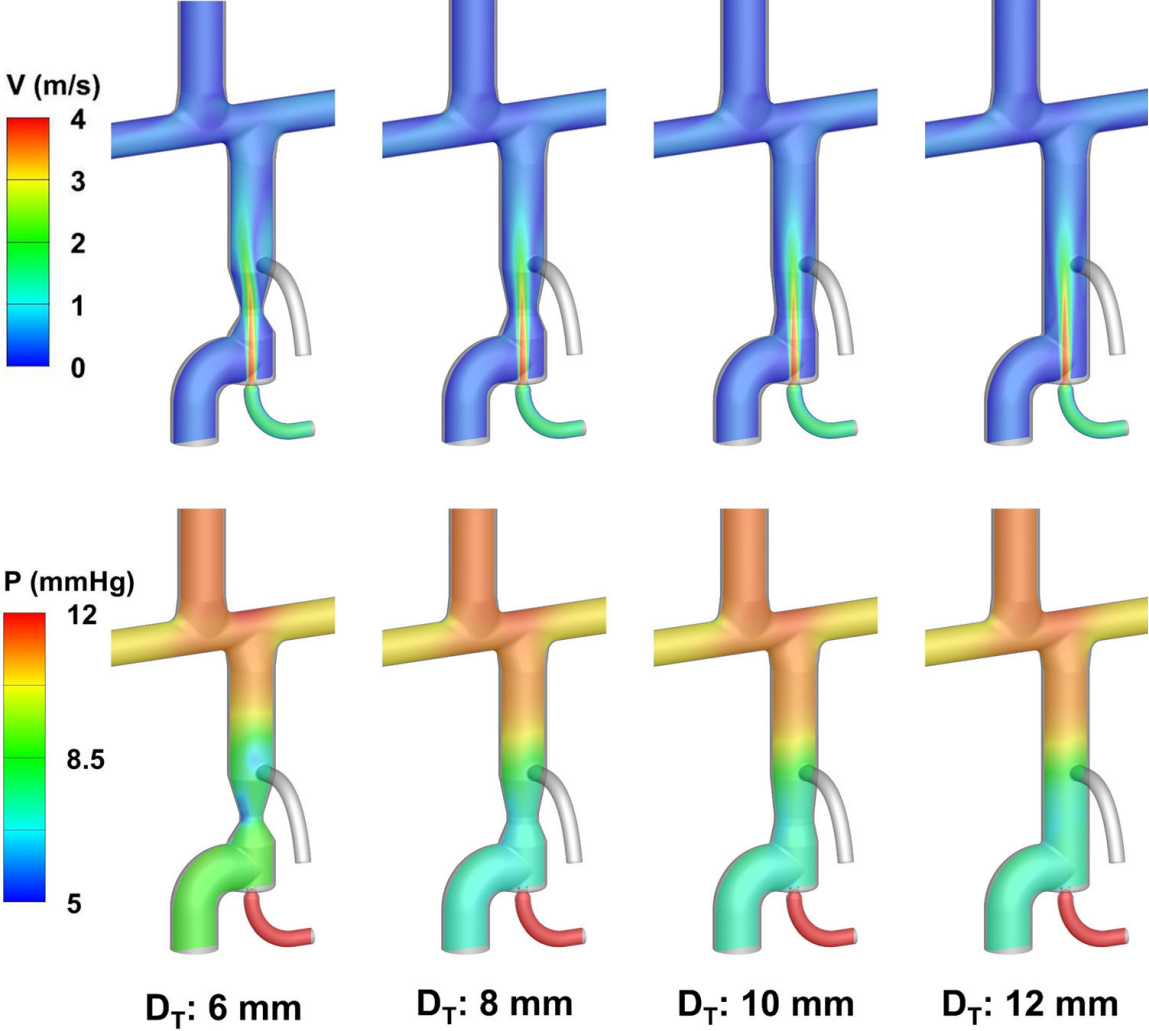
Impact of throat size on the velocity and pressure field in the one-diameter inlet offset idealized TCPC model. The color map represents the velocity magnitude (top) and pressure (bottom). The atrial discharge diameter is 4 mm.

**Figure 11:**
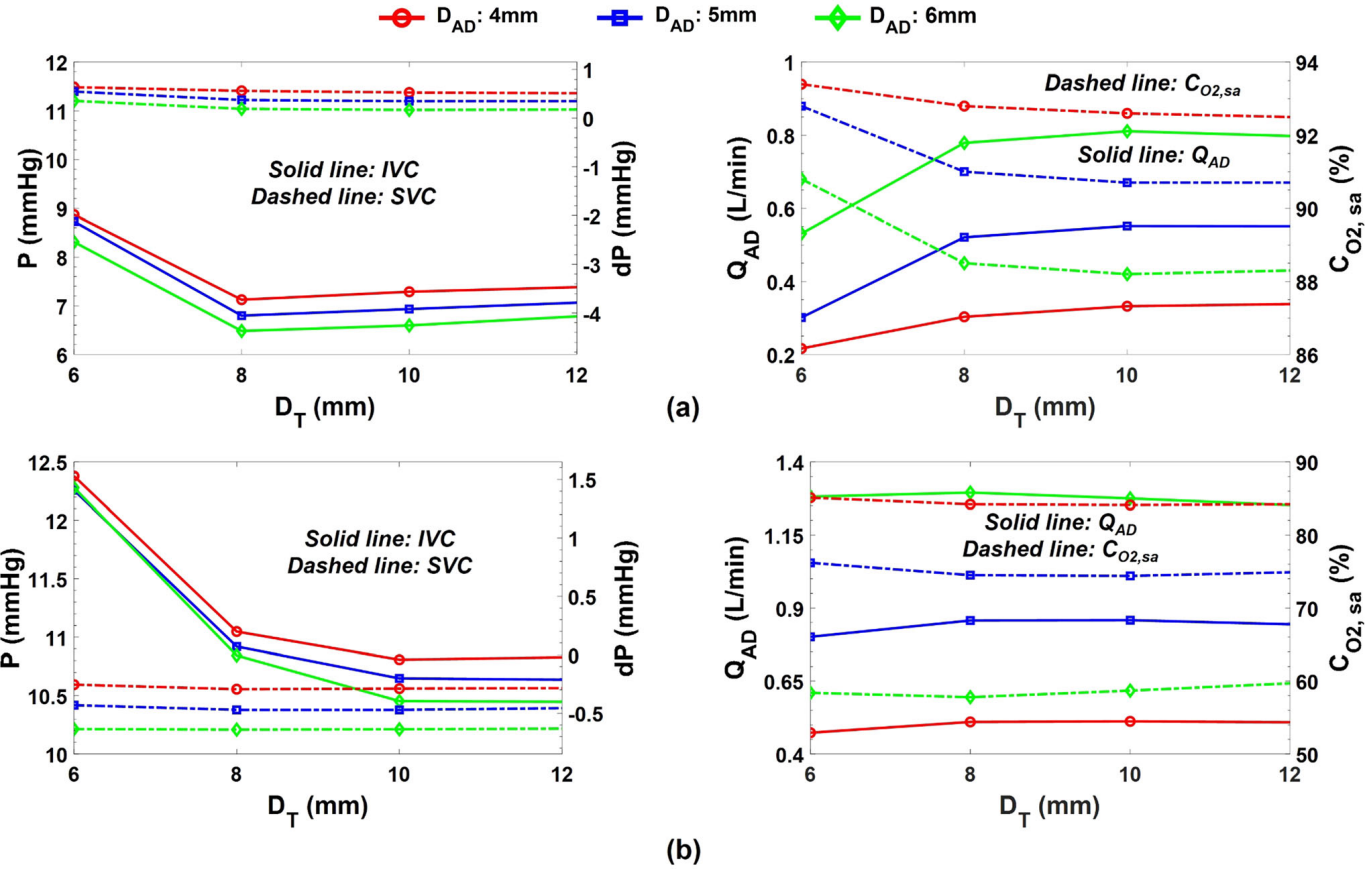
Impact of the throat and atrial discharge diameters on the key Fontan hemodynamic parameters in one-diameter inlet offset idealized TCPC model during (a) full assist and (b) failure of device scenarios. The nozzle diameter is 2.5 mm in these simulations, which provided the most significant IVC pressure drop while meeting the aortic graft flow criterion (CO/Q_S_ ≤ 1.5). *P* pressure, *dP* pressure change as compared to baseline, *D*_*T*_ throat diameter, *D*_*AD*_ atrial discharge diameter, *IVC* inferior vena cava, *SVC* superior vena cava, *Q*_*AD*_ atrial discharge flow rate, *C*_*O2,sa*_ systemic arterial oxygen saturation, *CO* cardiac output, and *Q*_*S*_ systemic flow rate. The baseline represents the TCPC state without the venous ejector pump.

Figure 10 shows the impact of throat diameter on the velocity and pressure field with an atrial discharge diameter of 4 mm. The key Fontan hemodynamic parameters for various throat and atrial discharge sizes are illustrated in Fig. 11. As discussed in Sect. "Impact of Aortic Nozzle Size", the nozzle diameter of 2.5 mm provided the greatest IVC pressure drop while meeting the aortic graft flow criterion (*Q*_AoG_/*Q*_S_ ≤ 0.5) and thus was selected in these simulations. The details of the simulation results are provided in the electronic supplementary material.

#### Performance During Device Full Assist Mode

Even though the level of pressure drops varied, all designs were successful at lowering the IVC pressure and maintaining a high systemic arterial oxygen saturation of more than 88%. Throats with diameters of 6 and 8 mm performed the worst and best in terms of IVC pressure drop, respectively. All cases experienced a slight increase in SVC pressure (less than 0.6 mm Hg), which was negatively correlated with the throat size, owing to higher atrial discharge flow rates at larger throat sizes. Narrower throats lead to a higher velocity at the throat, resulting in lower pressure and, as a result, a smaller pressure gradient between the atrium and the VEP, and thus lower atrial discharge flow rates. Furthermore, larger atrial discharge diameters provided higher IVC pressure drop and lower SVC pressure elevation.

#### Performance During Device Failure

A significant IVC pressure rise of 1.53 mm Hg was observed during aortic graft occlusion for the 6 mm throat. In the 8 mm throat, this was less than 0.2 mm Hg and negative in the other sizes. Low levels of oxygen saturations were observed for the 5 mm and 6 mm atrial discharge diameters (< 80%) during aortic graft occlusion. All cases generated a negative SVC pressure change as compared to the baseline condition (no VEP) during the aortic graft occlusion. The full failure of device (occlusion of both atrial discharge and aortic graft) resulted in an elevated IVC pressure of 0.59 mm Hg, 0.34 mm Hg and, 0.18 mm Hg for 8 mm, 10 mm and 12 mm throat sizes, respectively, as compared to baseline TCPC condition. No SVC pressure elevation was observed for the device’s full failure.

### Device Performance in Idealized and Patient-Specific Models

**Figure 12:**
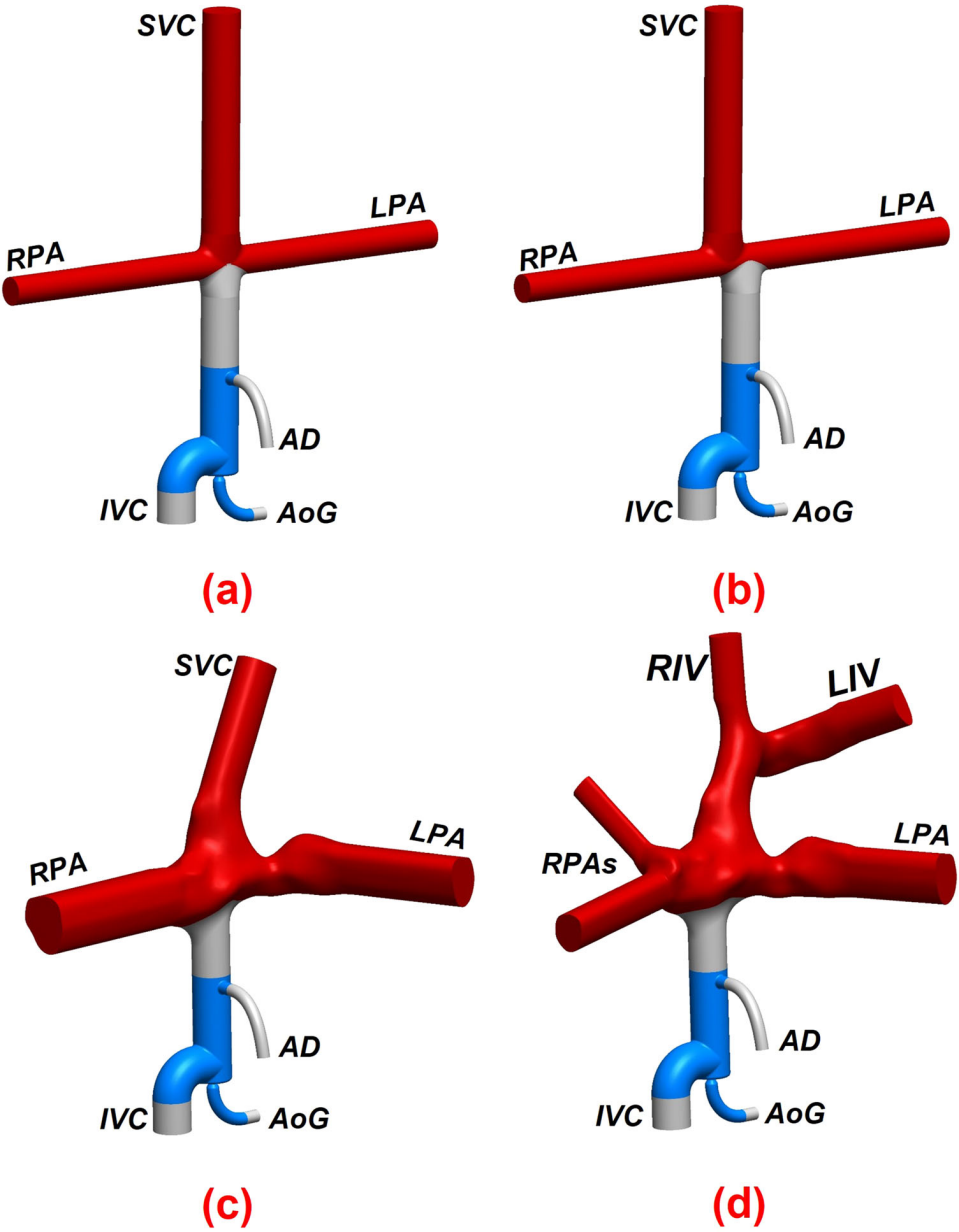
3D geometries of the idealized and patient-specific total cavopulmonary connections assisted by the optimal self-powered venous ejector pump (VEP). (a) 0 DO idealized TCPC, (b) 0.5 DO idealized TCPC, (c) patient-specific case 1 (PSC1) and, d) patient-specific case 2 (PSC2). The selected optimal VEP has an aortic nozzle diameter of 2.5 mm, throat size of 12 mm and, atrial discharge diameter of 4 mm. Red, blue, and grey colors represent the native vessels, the venous ejector pump, and the connecting grafts, respectively.

Based on the results presented in Sect. "Impact of the Throat and Atrial Discharge Size", one design prototype was selected with the geometrical parameters as stated below:

- *D*_N_: 2.5 mm, *D*_T_: 12 mm, *D*_AD_: 4 mm

where *D*_N_, *D*_T_, and *D*_AD_ represent the aortic nozzle, throat, and atrial discharge diameters, respectively. This design was found to provide significant IVC pressure drop during full assist while meeting the four criteria discussed in the Sect. "Geometrical Design Parameters". The selected VEP was then applied to the idealized TCPC models with zero and half-diameter inlet offsets, as well as patient-specific geometries, as shown in Fig. 12.

#### Performance During Device Full Assist Mode

**Figure 13:**
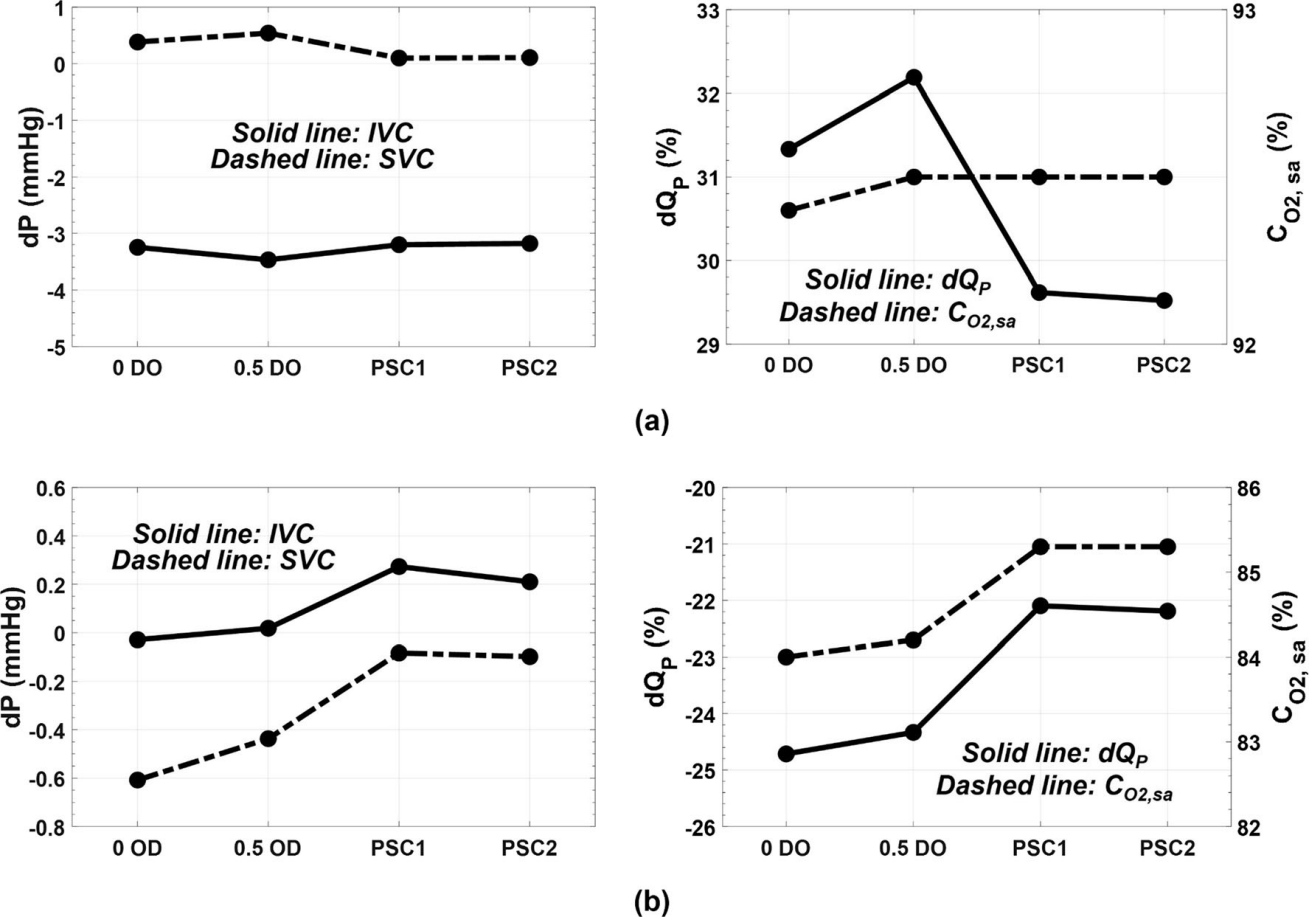
Performance parameters for the ideal VEP prototype in idealized and patient-specific TCPC models during (a) full assist and (b) failure of device scenarios. The ideal VEP has a throat and atrial discharge diameter of 12 mm and 4 mm, respectively. The aortic nozzle diameter is 2.5 mm in the prototypes. *dP* the pressure change as compared to baseline, *dQ*_*P*_ the change in pulmonary flow as compared to baseline, *IVC* inferior vena cava, *SVC* superior vena cava, *C*_*O2,sa*_ systemic arterial oxygen saturation, *0 OD* idealized TCPC model with zero diameter inlet offset, *0.5 OD* idealized TCPC model with 0.5 diameter inlet offset, *PSC1* patient-specific case 1, and *PSC2* patient-specific case 2. The baseline represents the TCPC state without the venous ejector pump. For PSC2, the SVC pressure represents the average pressure of the right and left innominate veins.

The performance of the VEP is depicted in Fig. 13 for both idealized and patient-specific models. The VEP was successful at maintaining a high systemic oxygen saturation of more than 90% and improving the pulmonary flow by more than 21%. An IVC pressure drop of 3.25 mm Hg (0 DO), 3.47 mm Hg (0.5 DO), and 3.2 mm Hg (patient-specific) was observed for the proposed VEP. A slight SVC pressure rise of less than 0.5 mm Hg was recorded in idealized models. The SVC pressure remained virtually unchanged (dP < 0.1 mm Hg) in the patient-specific models.

#### Performance During Device Failure Mode

Occlusion of aortic graft resulted in an IVC pressure increase of less than 0.24 mm Hg and systemic arterial oxygen concentration of higher than 84% for all models. Due to the presence of the atrial discharge, the pulmonary flow experienced a 22% reduction in flow rate in case of aortic graft occlusion in the patient-specific models. The SVC pressure change remained negative compared to the baseline (no VEP) condition for all models.

## Discussion

The performance of a proposed self-powered Fontan venous assist device is evaluated in this study using extensive computational fluid dynamics simulations. The device maintained a high systemic oxygen saturation of more than 90% while providing a significant IVC pressure drop of higher than 3.2 mm Hg for idealized and patient-specific models. These values compete with the existing percutaneous assist devices^38,55^ which use externally powered pumps that are prone to powerline infections. It's worth noting that nearly one-third of pediatric ventricle assist devices have led to serious complications due to powerline infections.^6^ The proposed venous ejector pump in this study eliminates the need for an external power source, allowing for better patient mobilization and rehabilitation while also preventing energy transmission-line infections, resulting in a higher quality of life for the patients.

The VEP demonstrated significant improvements to the Fontan hemodynamics with an aortic steal of around 30% of CO which is considered moderate when compared to aortopulmonary shunts with aortic steals as high as 50% used during the first stage of univentricular heart palliation.^49^ These levels of aortic steals exert a minor load on the ventricle which is well tolerated by single-ventricle patients in the long term. By incorporating the VEP into the Fontan circulation, both idealized and patient-specific TCPC models experienced a more than 21% increase in pulmonary flow, a significant drop in IVC pressure of more than 3.2 mm Hg, and systemic oxygen saturation of more than 90%. More importantly, in the patient-specific model, the SVC experienced no pressure change and was insignificant (0.3 mm Hg) in idealized cases, owing to the presence of atrial discharge. We speculate that by utilizing modified grafts such as Y-shaped extracardiac baffles^41^ for device-to-pulmonary anastomosis, a negative SVC pressure change can be achieved due to low levels of IVC/SVC flow impingement which can also lead to even more IVC pressure drop. This would also provide a balanced hepatic flow distribution to both lungs, preventing pulmonary arteriovenous malformations,^4,44,48^ a serious complication that can result in decreased systemic oxygen saturation after the Fontan. Because of the passive nature of VEP, an assisted extracardiac conduit incorporating VEP as well as a Y-shaped graft for device-to-pulmonary connection can be considered as the third stage of single-ventricle palliation instead of the conventional Fontan procedure. This way, a chronically low IVC pressure and an even distribution of the hepatic factor to pulmonary arteries could be achieved, resulting in a significantly improved cavopulmonary flow. Although sound hemodynamic improvements were achieved using the proposed prototype, the VEP design still has room for further modifications. The location of atrial discharge, more optimized throat geometry, and the possibility of accommodating SVC flow into the device are some of the intriguing design aspects which will be addressed in future works.

The previously suggested self-powered Fontan venous assist strategies lack either clinical feasibility or pose a high postoperative risk due to their complex structure and more importantly during the event of device failure. The systemic-to-pulmonary injection shunt proposed by Ni et al.^46^ failed to provide meaningful IVC pressure drop at constant pulmonary vascular resistances. The same concept, but with an injection shunt placed into the IVC extracardiac lumen rather than pulmonary arteries,^50^ was reported to provide 3.2 mm Hg IVC pressure drop but with a systemic oxygen saturation close to 80%. However, the primary cause of IVC pressure drop was the presence of a relatively large fenestration hole, and the inclusion of the injection shunt only provided an additional 0.2 mm Hg IVC pressure drop, as reported by the authors. Such large fenestration holes can lead to extremely low systemic oxygen saturation in the event of shunt occlusion. This issue is not a concern in the VEP since high systemic oxygen levels (≥ 84%) were achieved in both idealized and patient-specific models in case of aortic graft occlusion (Fig. 13b). More importantly, the unsupported injection shunt may experience displacement due to high momentum aortic flow which necessitates further considerations. An integrated turbine-pump system proposed by Pekkan et al.^47^ also showed venous pressure drop of 3 mm Hg during pediatric operation conditions in a pulsatile in vitro mock loop. However, aortic flow steals of lower than 0.9 L/min resulted in a negative net venous pressure augmentation. This would aggravate the IVC hypertension in case of a slight aortic graft occlusion due to thrombus formation. This is not a problem in the VEP since any amount of aortic flow steal lowers the IVC pressure to varying degrees. The failure of VEP with complete aortic graft blockage resulted in less than 0.2 mm Hg caval pressure rise in all TCPC models, underscoring the VEP’s fail-safe feature. The pump-turbine coupling and the bearing leakage is a challenging problem in these systems that has to be addressed. More importantly, the pulmonary flow pulsatility is improved during VEP operation due to the presence of aortic flow with a high pulsatility index (see electronic supplementary material). This issue is specifically important since a non-pulsatile pulmonary artery flow is believed to cause pulmonary hypertension and elevated pulmonary vascular resistance by disrupting endothelial functionality,^51,61^ leading to more severe IVC hypertension.

This study is subject to some limitations. One of the limitations is the assumption of the rigid wall for the vessels and the Fontan conduit in the computational simulations. Although the compliant nature of the veins and arteries can contribute to a more complex cavopulmonary flow profile, earlier studies have reported good agreement between the 4D in vivo data and the rigid wall simulations.^20,60^ Moreover, the time-averaged Fontan hemodynamic was recently shown to be unaffected by the wall compliance in a fluid–structure interaction simulation study,^62^ which further confirms the validity of the rigid wall assumption in Fontan computational simulations. The other limitation of this study is the assumption of a non-pulsatile (steady-state) condition for the caval inlet flows. Even though in vivo blood flow is pulsatile, the lack of subpulmonary ventricular flow in Fontan patients' total cavopulmonary connection leads to low levels of pulsatility in both the pulmonary arterial and systemic venous beds,^29^ highlighting the validity of the nonpulsatile caval flow assumption. Furthermore, earlier studies focused on Fontan computational simulations have shown good statistical agreement between using time-averaged and pulsatile boundary conditions.^17,21^ The respiration-induced caval flow pulsatility showed a positive impact on the VEP time-averaged performance (see electronic supplementary material). Another limitation is the aortic pressure pulsatility which was neglected in this study; however, our computational simulations revealed no significant difference between time-averaged device performance indices using a pulsatile and steady aortic pressure (see electronic supplementary material). Local Fontan hemodynamics evaluations were conducted in this study. Multi-scale computational modeling that includes vascular resistance and compliance coupled with the lumped parameter network will be required to better simulate the global hemodynamics of human circulation. Finally, the blood was assumed to be Newtonian. Albeit a non-Newtonian model was reported to alter the Fontan hemodynamic parameters,^8,9^ the impact of shear-thinning blood viscosity on the VEP performance is expected to be trivial due to the presence of high-velocity aortic jet flow.

The authors also acknowledge that the proposed venous ejector pump is still conceptual and further in silico as well as in vitro randomized patient-specific studies are warranted to evaluate the performance under both physiological and off-design conditions. The coronary artery perfusion pressure (CPP) is a crucial hemodynamic parameter in patients with a left-to-right shunt. A diastolic pressure higher than 30 mm Hg has been suggested to be an acceptable limit.^43^ The aortic graft in the proposed device resembles a central aortopulmonary shunt that draw blood from the ascending aorta to the pulmonary arteries. Previous computational patient-specific studies for central aortopulmonary shunts with 4 mm diameter (identical to the aortic graft inlet size in our study) have reported a coronary artery pressure of 36 mm Hg which is higher than the acceptable limit and thus myocardial ischemia and infarction due to reduced coronary artery pressure is less likely.^49^ It is worth mentioning that the shunt cross-section in the aforementioned study was constant which leads to significantly higher flow in the shunt compared to aortic graft in the present study with a restricting aortic nozzle of 2.5 mm. Thus, higher CPP values are expected during the operation of the VEP. Furthermore, the characterization of thrombogenicity and blood damage due to the presence of a left-to-right shunt with a small cross section is one of the most serious concerns for the VEP. Anticoagulant therapies, on the other hand, may reduce the risk of thrombus formation. More importantly, the proposed palliation relies on a functional single ventricle. Patients with an impaired ventricle will not benefit from the solution, and ventricle assist devices or cardiac transplantation remain the best options. In case of ventricular impairment after the VEP implantation, we speculate that percutaneous septal occlusion techniques can be utilized to close the aortic graft, resulting in the TCPC state circulation. The vascular dimensions and flow conditions used in this study corresponds to the small children; however, the proposed device has shown similar performance in conditions simulating younger adult (see electronic supplementary material). In the further development of the ejector pump, central issues will be strategies to prevent the clotting of blood and possibilities for percutaneous adjustments of the pump’s different dimensions. A pulsatile single-ventricle circulatory mock loop is currently being developed in our laboratory to examine the in vitro performance of the device which will be presented in future communications.

## Conclusion

A self-powered venous ejector pump is proposed in this study for Fontan patients suffering from elevated IVC pressure. The proposed solution is clinically feasible, simple in structure, and powered intracorporeally, eliminating the need for an external power source and driveline infection-associated complications. Extensive computational fluid dynamics simulations were conducted to evaluate the performance in both idealized and 3D reconstructed patient-specific TCPC models. Significant IVC pressure drop of more than 3.2 mm Hg were observed for both the idealized and patient-specific TCPC models, with a moderate aortic steal of around 30% of CO while maintaining high levels of systemic oxygen saturation of more than 90%, demonstrating its potential to provide palliation for the growing population of patients with failing Fontan.

## Supplementary Information

Below is the link to the electronic supplementary material.

Supplementary file1 (PDF 2180 kb)
