# Supplementary material for: In Silico Evaluation of a Self-powered Venous Ejector Pump for Fontan Patients
Source: Cardiovasc Eng Technol. 2023 Mar 6;14(3):428–46. doi: 10.1007/s13239-023-00663-5 (PMC10412470; doi:10.1007/s13239-023-00663-5)
Supplement: Supplementary file 1 — Supplementary file1 (PDF 2180 kb) [file 13239_2023_663_MOESM1_ESM.pdf]

Supplementary Material

In Silico Evaluation of a Self-Powered Venous Ejector Pump for Fontan Patients

Reza Rasooli, Knut Erik Teigen Giljarhus, Aksel Hiorth, Ingunn Westvik Jolma, Jan Ludvig Vinningland, Charlotte de Lange, Henrik Brun, Henrik Holmstrom

1. Impact of aortic pulsatility and CFD model on the VEP performance

In order to investigate the impact of aortic pulsatility on the device performance, one case (VEP with 1 DO TCPC) was simulated using a pulsatile aortic pressure. A physiological aortic pressure waveform with a mean aortic pressure and pulse pressure of 51.1 mm Hg and 40 mm Hg, respectively, was considered for the aortic graft inlet boundary condition. The aortic graft total pressure was assigned as the boundary condition. Unsteady RANS with the same computational schemes described in the main text was utilized. A second-order temporal discretization with a time step of 1 ms was considered. A residual reduction of e-4 was considered for the inner iterations. Figure S1.1 depicts the temporal variations of the Fontan hemodynamics with a pulsatile aortic pressure. Table S1.1 compares the hemodynamic indices for time-averaged pulsatile and steady time-averaged aortic pressure boundary conditions. As it can be observed, the device performance strongly correlates with the mean flow characteristics thus showing the validity of a time-averaged aortic boundary condition. More importantly, this result reveals the ability of the VEP to significantly increase the pulmonary arterial pulsatility which is believed to improve the pulmonary vascular health and reduce the pulmonary vascular resistance. Moreover, to examine the impact of implemented CFD model on the time-averaged device and Fontan hemodynamic indices, the same physics was simulated using large eddy simulation (LES) CFD model with the settings similar to reference [1]. Figure S1.2 and Table S1.2 present the device performance and Fontan hemodynamic metrices for the two CFD models. No significant difference is observed

between time-averaged values showing the validity of using Reynolds averaged turbulence models.

Table S1.1. Comparison of hemodynamic indices for pulsatile and steady aortic pressure condition. The pulsatile data represent the time-averaged value over 2 cardiac cycles.

	Pulsatile	Steady
**Q_{AD} (L/min)**	0.54	0.54
**P_{AoG} (mm Hg)**	51.1	51.1
**P_{IVC} (mm Hg)**	8.63	8.70
**Q_P (L/min)**	2.52	2.53
**P_{SVC} (mm Hg)**	11.32	11.32

Table S1.2. Impact of aortic pressure pulsatility on the VEP performance and Fontan hemodynamic indices using two CFD models. Q_{AD}: atrial discharge flow, P_{IVC}: IVC pressure, Q_P: pulmonary flow, P_{SVC}: SVC pressure, StAoP: steady aortic pressure, PAoP: pulsatile aortic pressure.

	**URANS SST K-ω (StAoP)**	**URANS SST K-ω (PAoP)**	**LES (StAoP)**	**LES (PAoP)**
**Q_{AD} (L/min)**	0.54	0.54	0.57	0.59
**P_{AoG} (mm Hg)**	51.5	51.5	51.9	51.9
**P_{IVC} (mm Hg)**	8.70	8.63	8.44	8.54
**Q_P (L/min)**	2.53	2.52	2.44	2.41
**P_{SVC} (mm Hg)**	11.32	11.32	11.23	11.2

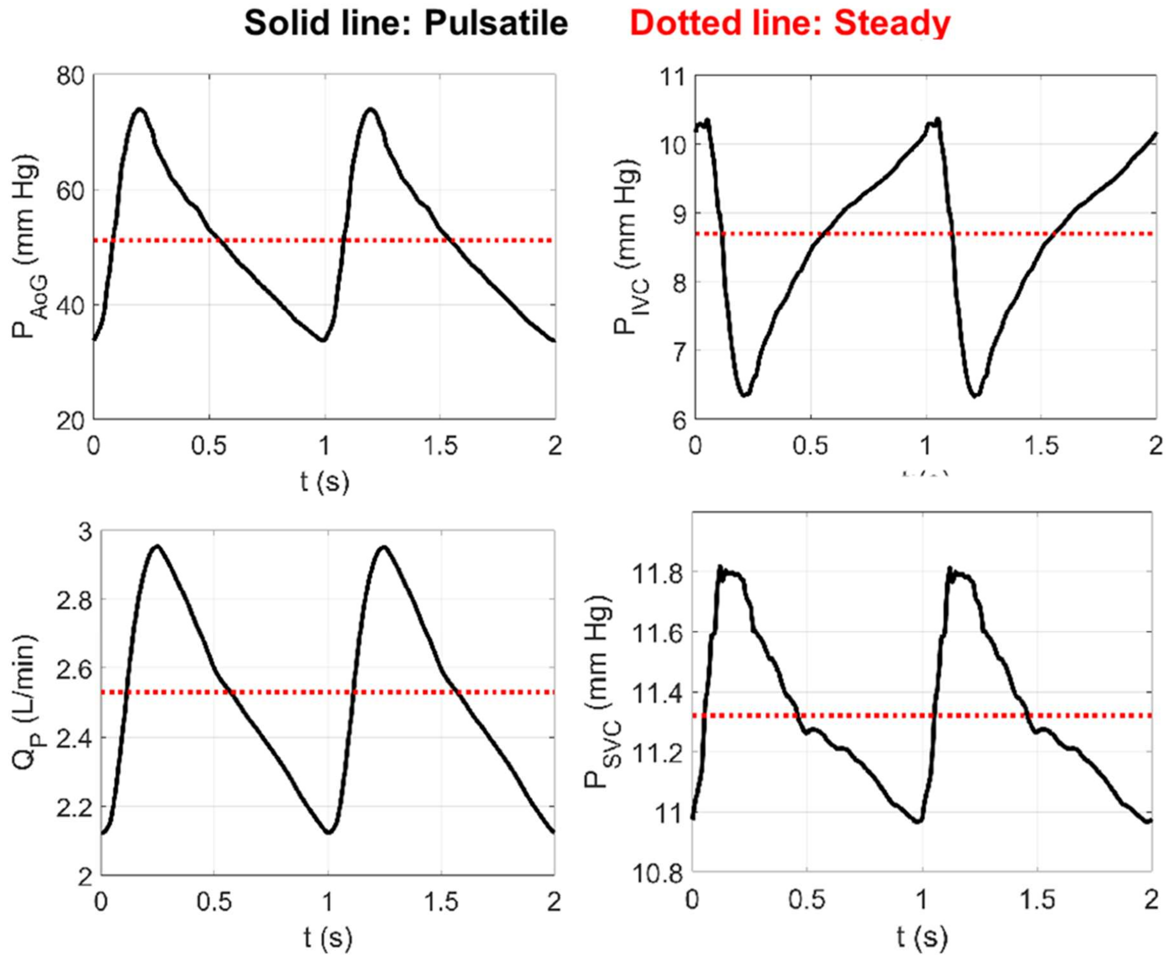

Figure S1.1. Impact of aortic flow pulsatility on the device's performance and Fontan hemodynamics with a pulmonary arterial pressure of 10 mm Hg. P_{AoG}: pressure at the aortic graft inlet, P_{IVC}: IVC pressure, P_{SVC}: SVC pressure, Q_P: pulmonary artery flow rate, Q_{AD}: atrial discharge flow rate.

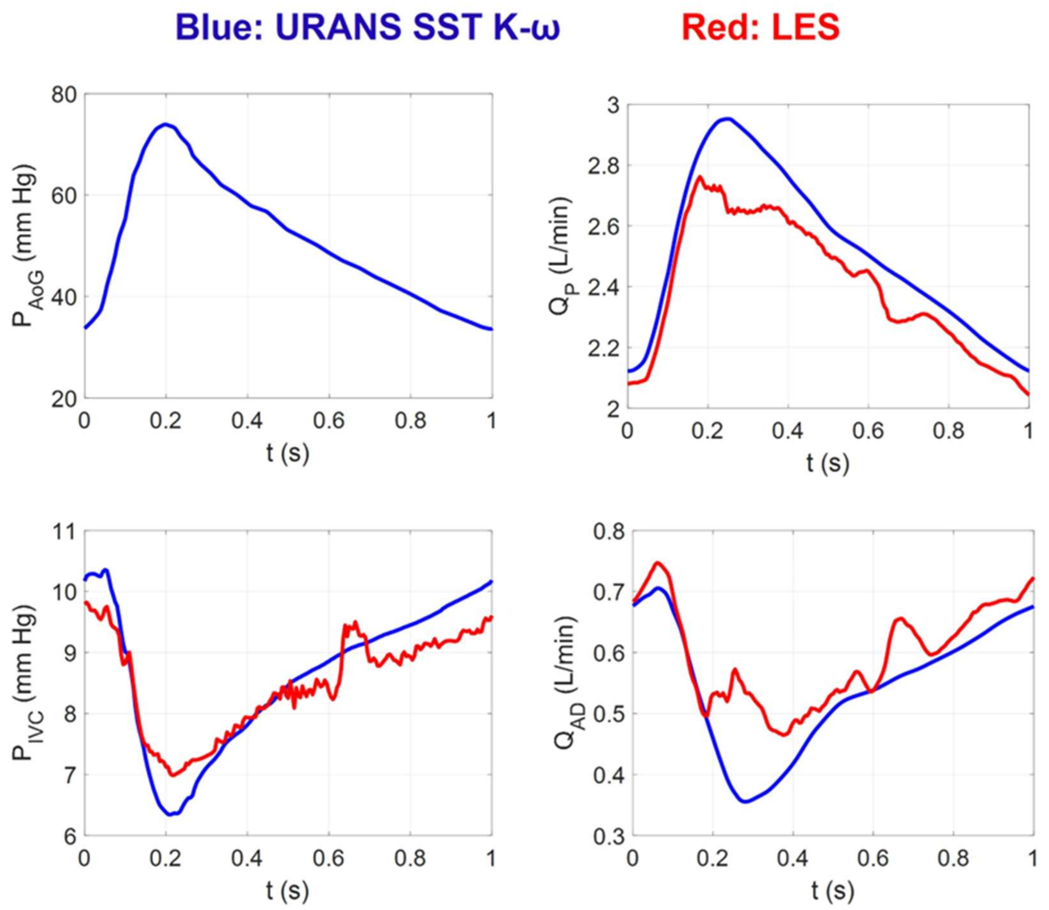

Figure S1.2. Impact of aortic pulsatility on the Device performance and Fontan hemodynamics using two CFD models.

2. CFD solver experimental validation

2.1. FDA nozzle benchmark validation

The exhaustion of the aortic nozzle jet in the VEP resembles the flow physics of the FDA nozzle jet expansion; however, the presence of a cross-flow from the IVC side and its interaction with the aortic jet as well as the atrial discharge contributes to a more complex and turbulent flow characteristic with the possible earlier transition. Therefore, the FDA nozzle with a Reynolds number of 6500 was selected as the most relevant case to account for a more turbulent flow profile in VEP. Figure S2.1.1 represents the centerline velocity variation predicted by the CFD solver settings used in this study as well as the available experimental data in the literature [2]. Good agreement can be observed between the CFD predicted flow and the experimental data for both the centerline velocity and the jet breakdown location.

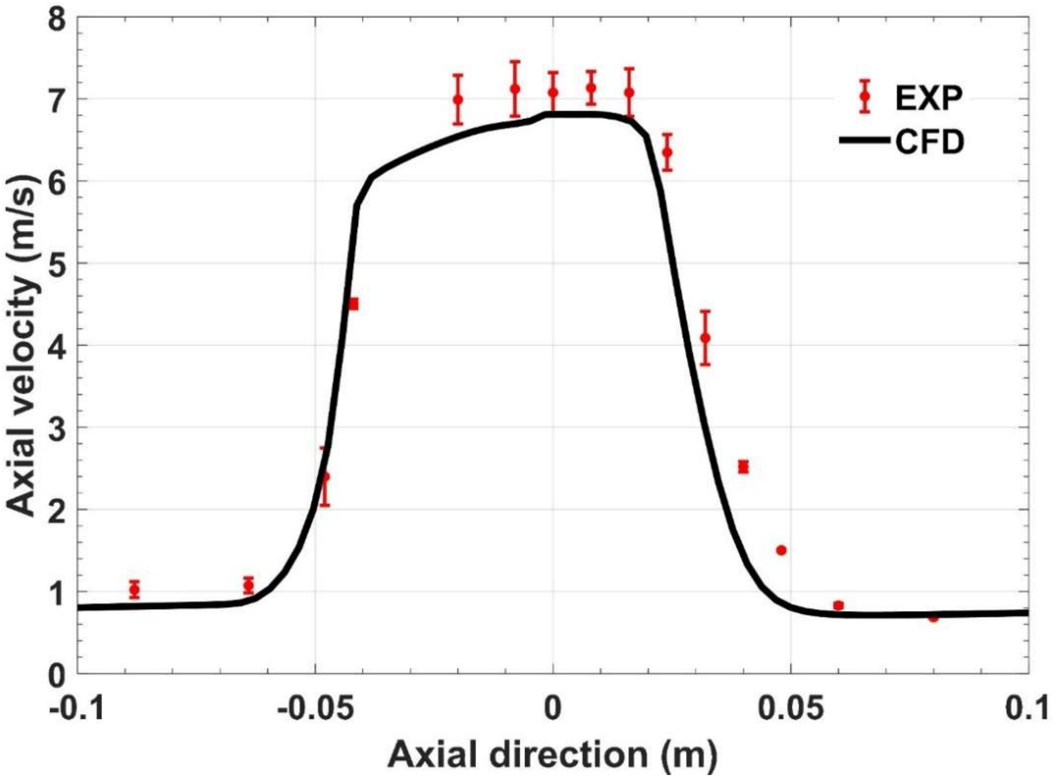

Figure S2.1.1 Comparison of axial velocity from CFD simulation and the available PIV data along the centerline of the benchmark FDA nozzle. The CFD represents the same solver settings used to asses the venous ejector pump performance in this study. The experimental data (EXP) are the average values taken from a multi-laboratory study [2]. The error bars represent the standard deviation from the average.

2.2. Device-specific pressure validation

To assess and validate the pressure prediction accuracy of the employed computational fluid dynamics solver settings, an experimental validation was conducted in our laboratory (Figure S2.2.2). The VEP with throat and aortic nozzle diameters of 8 mm and 2.5 mm, respectively, and no atrial discharge was considered for this experiment. The protype was 3D printed using stereolithography (Form 3, Formlabs, Somerville, MI, USA). A magnetically coupled centrifugal pump (Xylem, Washington, USA) was used to generate steady flow to the IVC and aortic graft. The flow into the IVC and aortic graft was adjusted using pinchcock tube clamp (Bochem instrumente GmbH, Weilburg, Germany) and was measured using clamp-on ultrasonic flowmeters (Sonotec GmbH, Halle, Germany). Tygon 3603 flexible tubing (Saint-Gobain, Courbevoie, France) was used for the connections. Water was used as the working fluid and the flowmeters were calibrated for the specific fluid and tubing prior to experiments. The outlet static pressure was kept constant at 10 mm Hg by using a 3D printed tank that keeps the height of the fluid constant. Non-invasive pressure transducers with luer fittings (BDC laboratories, Colorado, USA) was used to record the pressure at the IVC inlet. The experiments were conducted at different IVC and aortic flow rates. The pressure drop compared to zero aortic flow condition was considered the parameter for validation to minimize the outlet pressure uncertainty. The experiments were conducted three times to ensure repeatability. Table S2.2.1 compares the experimental data with the CFD simulations predictions. The computational scheme and settings were identical to the one in the manuscript. The experimental data are the average of three experiments and the numbers in paranthesis represent the amplitude of fluctuations. As it can be observed, the employed CFD settings is successful at providing acceptable prediction for the pressure accuracy.

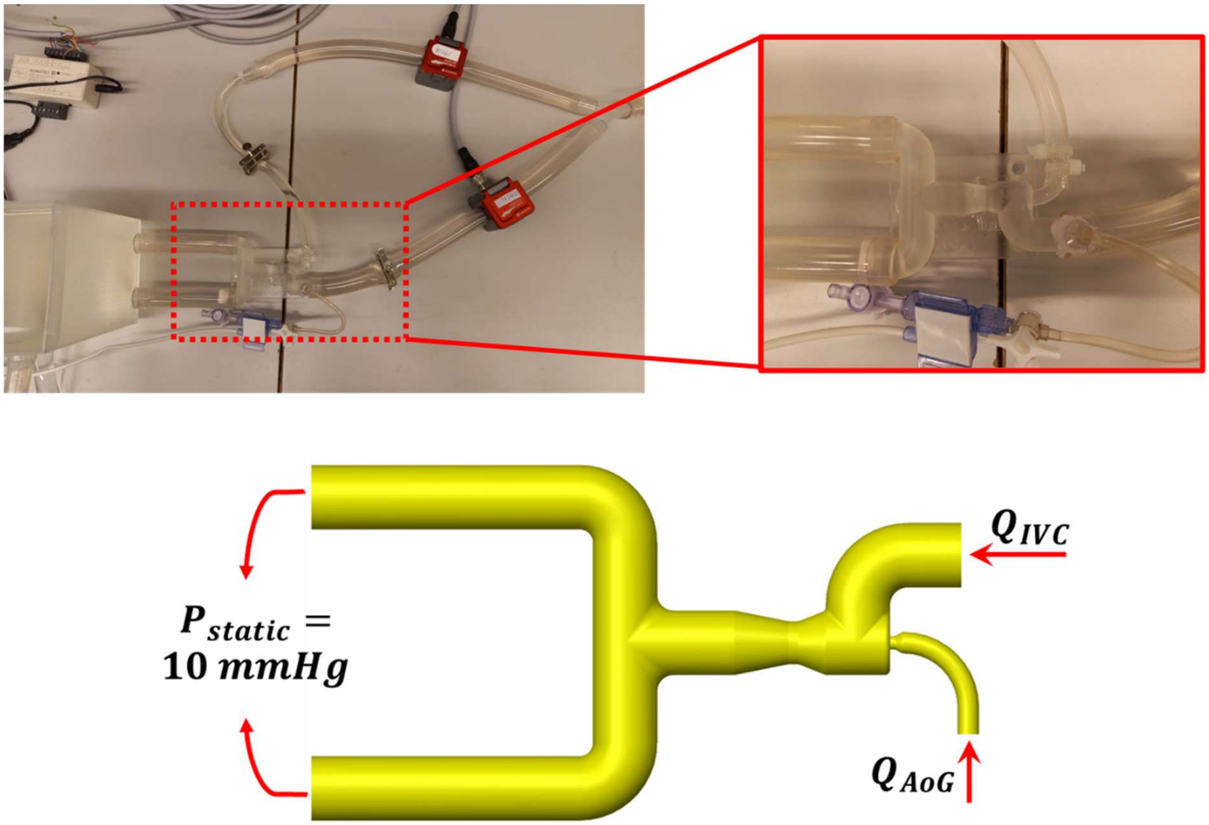

Figure S2.2.1 Experimental setup and boundary conditions for the validation experiment. IVC: inferior vena cava, AoG: aortic graft.

Table S2.2.1 Comparison of CFD predicted pressure drop and experimental data. The experimental values are the average of three experiments and the numbers in the paranthesis represent the amplitude of fluctuations. The pressure drop (dP) represents the magnitude of pressure change compared to the case with zero aortic graft flow.

**Q (L/min)**		**CFD**	**EXP**
IVC	AoG	dP (mm Hg)	dP (mm Hg)
0.5	0.5	1.11	1.06±0.06 (0.6)
0.5	1.0	5.32	5.13±0.1 (1.7)
1.0	0.5	0.64	0.77±0.1 (0.8)
1.0	1.0	4.33	4.10±0.1 (1.7)

3. Device performance evaluation in younger adult patients

In order to evaluate the performance and capabilities of the proposed venous ejector pump in different patient groups, computational simulations were conducted using relevant vascular dimesnions and pressure conditions. For this purpose, an idealized TCPC with one diameter inlet offset with IVC and SVC vessel diameter of 16 mm and pulmonary arterial diameter of 12 mm was constructed using Autodesk inventor (Autodesk, California, USA). Moreover, the patient-specific case 1 utilized in the main text was also reconstructed with a 16 mm IVC conduit. The cardiac output was considered as 4.2 L/min with an IVC/SVC flow ratio of 60/40. The mean aortic pressure (MAP) and pulmonary arterial pressure were assigned as 93 mm Hg and 14 mm Hg, respectively, to simulate a hypertensive condition in young adults. The Table S4.1 summarizes the results for the scaled VEP proposed in the manuscript. The scaling was applied only to accommodate the desired IVC size. Atrial discharge and aortic graft geometrical designs and dimensions are kept identical to the ones proposed in the manuscript (Figure S3.1). As it can be observed, the device can provide significant IVC pressure drop of 3.1 mm Hg and ~3 mm Hg in idealized and patient-specific models, respectively, as well as excellent systemic oxygen concentration in these patient groups. The beauty of the proposed design is its simplicity and the fact that it can be readily 3D printed using surgical and bio-compatible materials with ultra-low costs based on patient size in any clinical centers or research laboratories. However, we acknowledge that the full spectrum of the blood damage characteristics and thrombogenicity has to be investigated and studied in great details which we believe is beyond the purview of the present manuscript and requires a separate dedicated study. The most important aspect of the design is that if the two grafts (atrial discharge and aortic) that are the most susceptible parts to thrombus formation get completely occluded, the circulation returns to its TCPC pre-VEP conditions with the minimal damage to the patient as evident in the Table S3.1.

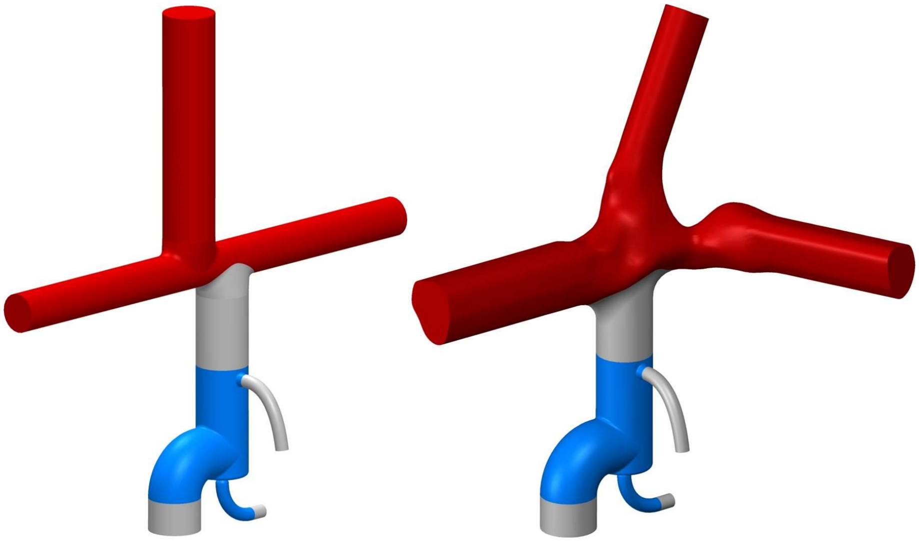

Figure S3.1. 3D geometry of scaled VEP integrated in (a) 1DO idealized and (b) patient-specific TCPC models with 16 mm IVC conduit size.

Table S3.1. Computational results for the proposed VEP simulating young adult condition. The IVC diameter, pulmonary artery diameter, mean aortic pressure, cardiac output and pulmonary arterial pressure are 16 mm, 12 mm, 93 mm Hg, 4.2 L/min and, 14 mm Hg, respectively. The IVC diameter for the patient-specific case 1 was considered to be 16 mm. The full assist represents no blockage in both atrial discharge and aortic graft. Full failure represents complete occlusion of both atrial discharge and aortic grafts. AoG blocked represents complete occlusion of aortic graft with an open atrial discharge. Q_{AD}: atrial discharge flow, Q_{AoG}: aortic graft flow, P_{IVC}: IVC pressure, Q_P: pulmonary flow, P_{SVC}: SVC pressure.

	**Full Assist**	**Full Failure**	**AoG Occluded**	**Full Assist**	**Full Failure**	**AoG Occluded**
	**Idealized 1DO**			**Patient-specific Case 1**		
**P_{IVC} (mm Hg)**	11.9	15.13	15.07	11.56	14.77	14.64
**P_{SVC} (mm Hg)**	15.35	15.04	14.8	14.61	14.42	14.29
**Q_{AoG} (L/min)**	1.26	0	0	1.25	0	0
**Q_{AD} (L/min)**	0.53	0	0.68	0.56	0	0.66
**Q_P (L/min)**	4.93	4.2	3.52	4.89	4.2	3.54
**$P_{IVC, Base}$ (mm Hg)**	15.00	15.00	15.00	14.56	14.56	14.56
**$P_{SVC, Base}$ (mm Hg)**	15.04	15.04	15.04	14.50	14.50	14.50
**dP_{IVC} (mm Hg)**	-3.10	0.13	0.07	-3.0	0.21	0.08
**dP_{SVC} (mm Hg)**	0.31	0	-0.24	0.11	-0.08	-0.21
**$C_{sa,O2}$ (%)**	92	95	80	91	95	80

4. Impact of respiration-induced pulsatility on the device performance

Although the caval flow in Fontan patients lacks pulsatility due to absence of a subpulmonary ventricle, respiration-induced pulsatility have been shown to be significant in these patient groups [3]. The pulsatility level is quantified using respiratory dependency (RD) parameter as [4]:

$$RD = 100 \times \frac{Q_{ins} - Q_{exp}}{Q_{ins} + Q_{exp}}$$

Where Q_{ins} and Q_{exp} are inspiratory and expiratory flow, respectively. It has been reported that the RD in IVC can reach as high as 200%, resulting in a retrograde flow in the IVC caval flow [3]. However, the RD level in SVC has been reported to be relatively small (<30%) compared to IVC caval flow. To understand the impact of respiration-induced pulsatility on the VEP performance, one case (VEP1 with 1 DO TCPC) was simulated using a pulsatile aortic pressure and respiration-induced pulsatile IVC flow rate. A physiological aortic pressure waveform with a mean aortic pressure and pulse pressure of 51.1 mm Hg and 40 mm Hg, respectively, was considered for the aortic graft inlet boundary condition. The aortic graft total pressure was assigned as the boundary condition. Unsteady RANS with the same computational schemes described in the main text was utilized. A second-order temporal discretization with a time step of 1 ms was considered. A residual reduction of e-4 was considered for the inner iterations. The cardiac output, IVC/SVC flow ratio and pulmonary arterial pressure values were identical to the ones used in the main text. SVC flow rate was considered non-pulsatile. Different sinusoidal IVC flow with averaged flow rate of 1.26 L/min, respiratory dependency (RD) of 30%, 80% , 200%, and respiration cycle of 4 s were considered to account for the respiratory-induced IVC flow, in line with previously reported patient-specific data [5]. The IVC flow waveform with 200% RD resulted in retrograde flow with retrograde to mean forward flow ratio of 18%, in agreement with the reported clinical data [6-8]. Figure S5.1 depicts the IVC flow rate over one respiratory cycle for different RD values. Previously discussed physiological aortic pressure waveform was used as the inlet boundary condition for the aortic graft inlet. Table S5.1 summarizes the time-averaged hemodynamic indices over one respiratory cycle. The first cycle of the simulations was ignored due to starting effects. Figure S5.2 depicts the instantaneous Fontan hemodynamic parameters for different respiration-induced flow pulsatility. As it can be observed, in addition to altered instantaneous hemodynamics, the time-averaged properties are also significantly changed with respiration-induced pulsatility. Interestingly, the IVC pressure reduction efficiency of the VEP has a negative correlation with the

respiration-induced pulsatility in which higher pulsatility levels resulted in lower IVC pressure and thus improved device performance. Therefore, the conclusions reached using no respiration-induced pulsatility in the manuscript are valid as the pulsatility improves the performance.

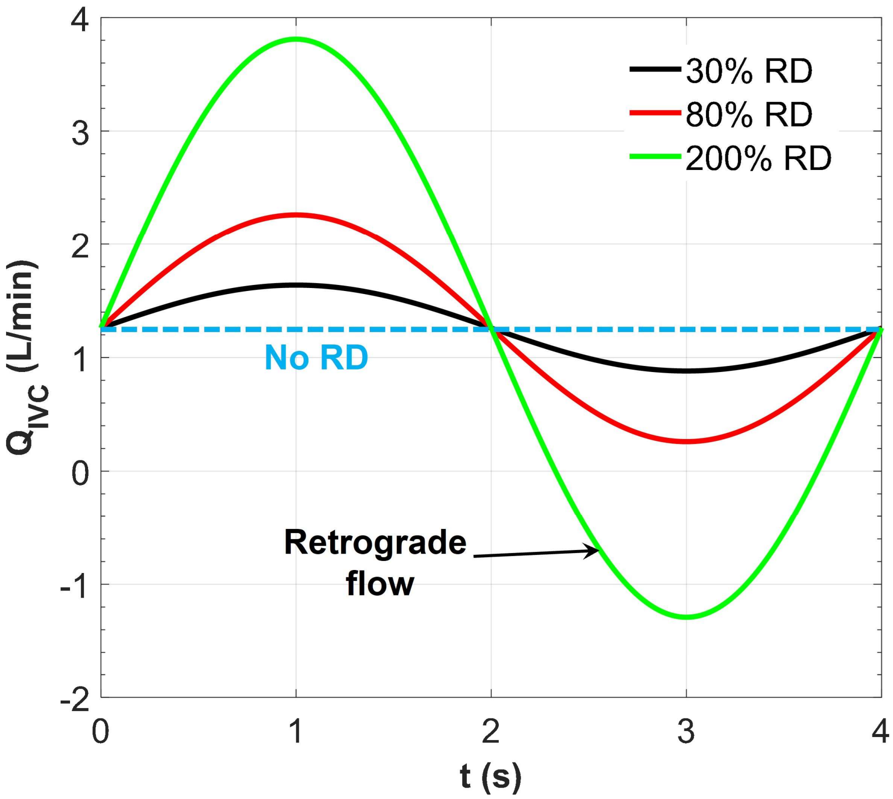

Figure S5.1. IVC flow rate with different respiration-induced pulsatility levels. RD: respiratory dependency.

Table S5.1. Impact of respiration-induced pulsatility level and retrograde flow on the time-averaged device performance and the Fontan hemodynamics. Q_{AD}: atrial discharge flow, Q_{AoG}: aortic graft flow, P_{IVC}: IVC pressure, Q_P: pulmonary flow, P_{SVC}: SVC pressure and, RD: respiratory dependency.

	200% RD (retrograde flow)	80% RD (No retrograde flow)	30% RD (No retrograde flow)	No RD
Q_{AD} (L/min)	0.73	0.62	0.56	0.54
Q_{AoG} (L/min)	0.97	0.97	0.96	0.96
P_{IVC} (mm Hg)	8.17	8.28	8.45	8.63
Q_P (L/min)	2.34	2.45	2.51	2.52
P_{SVC} (mm Hg)	11.48	11.31	11.34	11.32

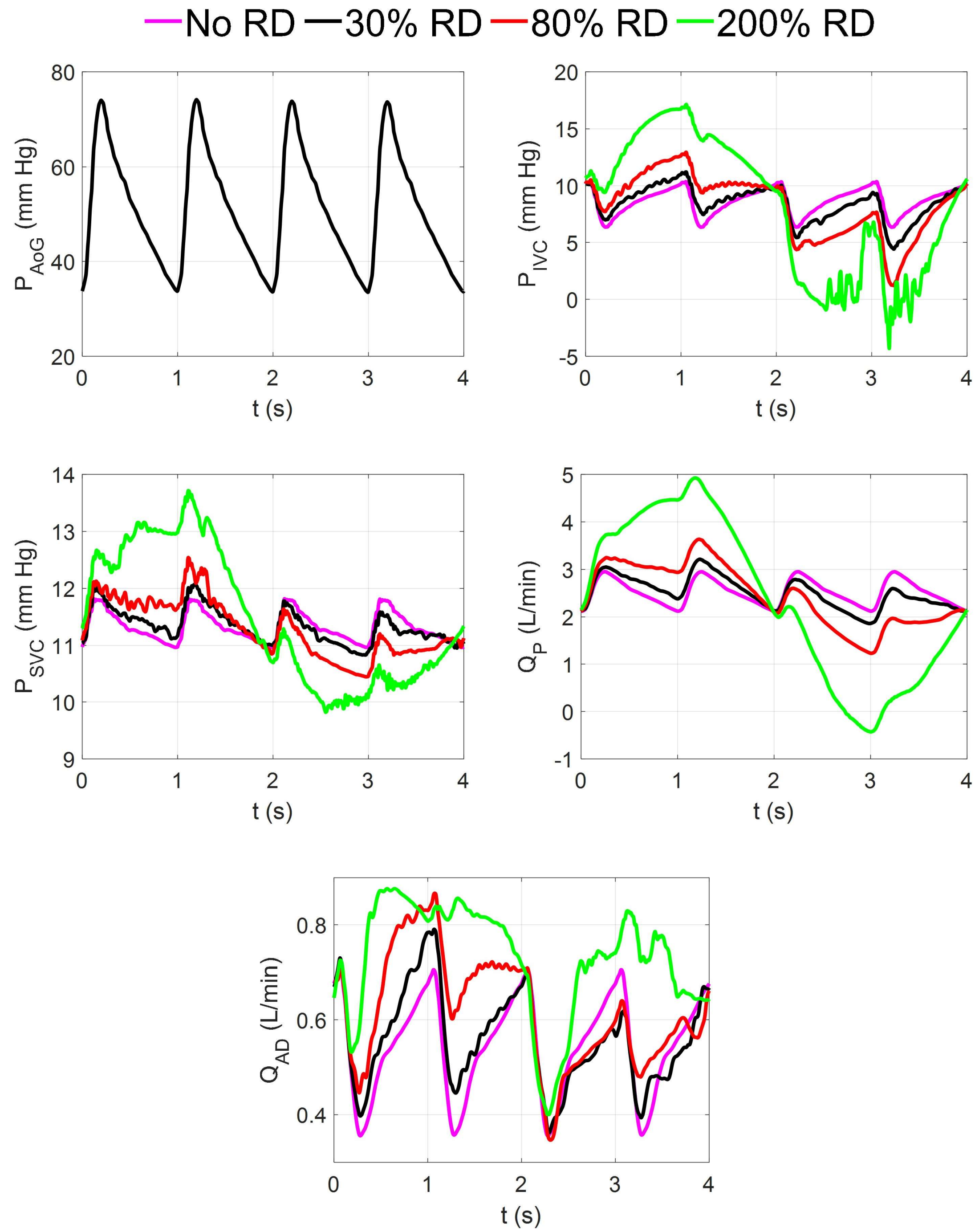

Figure S5.2. Impact of respiration-induced IVC flow pulsatility on the VEP performance and the Fontan hemodynamics. Q_{AD}: atrial discharge flow, P_{AoG}: aortic pressure waveform, P_{IVC}: IVC pressure, Q_P: pulmonary flow, P_{SVC}: SVC pressure and, RD: respiratory dependency.

5. Details of CFD simulation data

In this section, the details of the computational results for all cases are reported.

Table S5.1. CFD simulation results for different aortic nozzle diameters. The baseline condition represent the case without VEP. IVC: inferior vena cava, SVC: superior vena cava, dP: pressure difference as compared to baseline condition, AoG: aortic graft, AD: atrial discharge, CO: cardiac output, Qs: systemic flow rate, Co2,sa: systemic arterial oxygen concentration, m: atrial discharge concentration variable as described in the main text.

	Baseline	**Nozzle diameter (mm)**			
		1.5	2.0	2.5	3.0
**P_{IVC} (mmHg)**	10.850	10.051	9.175	7.201	4.360
**P_{SVC} (mmHg)**	10.868	10.760	11.031	11.355	11.970
**dP_{IVC} (mmHg)**	--------	-0.799	-1.675	-3.649	-6.490
**dP_{SVC} (mmHg)**	--------	-0.108	0.163	0.487	1.102
**Q_{AoG} (L/min)**	--------	0.315	0.597	0.993	1.555
**Q_{AD} (L/min)**	--------	0.430	0.349	0.318	0.219
**CO/Q_S**	1	1.150	1.284	1.472	1.741
**$C_{O2,sa}$ (%)**	95	89	91	93	94
**m**	--------	0.1980	0.3215	0.4408	0.5524

Table S5.2. CFD simulation results for the full assist mode of the venous ejector pump using idealized 1-DO TCPC model with different throat and atrial discharge diameters. D_T: throat diameter, D_{AD}: atrial discharge diameter, IVC: inferior vena cava, SVC: superior vena cava, dP: pressure difference as compared to baseline condition, AoG: aortic graft, AD: atrial discharge, CO: cardiac output, Qs: systemic flow rate, $C_{o2,sa}$: systemic arterial oxygen concentration, m: atrial discharge concentration variable as described in the main text, Q_P: pulmonary flow rate.

	**D_T: 6mm**			**D_T: 8mm**			**D_T: 10mm**			**D_T: 12mm**		
**D_{AD} (mm)**	**4**	**5**	**6**	**4**	**5**	**6**	**4**	**5**	**6**	**4**	**5**	**6**
**P_{IVC} (mmHg)**	8.869	8.730	8.311	7.124	6.797	6.481	7.288	6.932	6.595	7.382	7.063	6.782
**P_{SVC} (mmHg)**	11.489	11.399	11.207	11.413	11.225	11.043	11.379	11.200	11.021	11.364	11.200	11.028
**dP_{IVC} (mmHg)**	-1.981	-2.120	-2.539	-3.726	-4.053	-4.369	-3.562	-3.918	-4.255	-3.468	-3.787	-4.068
**dP_{SVC} (mmHg)**	0.621	0.531	0.339	0.545	0.357	0.175	0.511	0.332	0.153	0.496	0.332	0.160
**Q_{AoG} (L/min)**	0.999	1.002	1.006	1.016	1.020	1.023	1.014	1.019	1.022	1.013	1.018	1.020
**Q_{AD} (L/min)**	0.216	0.301	0.531	0.303	0.521	0.779	0.332	0.552	0.811	0.338	0.551	0.798
**Q_P (L/min)**	2.883	2.801	2.575	2.813	2.599	2.344	2.782	2.567	2.311	2.775	2.567	2.322
**CO/Q_S**	1.476	1.477	1.479	1.484	1.486	1.487	1.483	1.485	1.487	1.482	1.485	1.486
**Q_P/Q_S**	1.373	1.334	1.226	1.340	1.238	1.116	1.325	1.222	1.100	1.321	1.222	1.106
**$C_{O2,sa}$ (%)**	93	93	91	93	91	89	93	91	88	93	91	88
**m**	0.4426	0.4536	0.4421	0.4523	0.4577	0.4493	0.4651	0.4539	0.4546	0.4545	0.4520	0.4505

Table S5.3. CFD simulation results for the case of occluded aortic graft using idealized 1-DO TCPC model with different throat and atrial discharge diameters. D_T: throat diameter, D_{AD}: atrial discharge diameter, IVC: inferior vena cava, SVC: superior vena cava, dP: pressure difference as compared to baseline condition, AoG: aortic graft, AD: atrial discharge, CO: cardiac output, Qs: systemic flow rate, $C_{o2,sa}$: systemic arterial oxygen concentration, m: atrial discharge concentration variable as described in the main text, Q_P: pulmonary flow rate.

	**D_T: 6mm**			**D_T: 8mm**			**D_T: 10mm**			**D_T: 12mm**		
**D_{AD} (mm)**	**4**	**5**	**6**	**4**	**5**	**6**	**4**	**5**	**6**	**4**	**5**	**6**
**P_{IVC} (mmHg)**	12.379	12.258	12.280	11.048	10.920	10.844	10.808	10.648	10.454	10.828	10.637	10.448
**P_{SVC} (mmHg)**	10.595	10.419	10.214	10.556	10.379	10.209	10.561	10.379	10.212	10.565	10.393	10.218
**dP_{IVC} (mmHg)**	1.529	1.408	1.430	0.198	0.070	-0.006	-0.042	-0.202	-0.396	-0.022	-0.213	-0.402
**dP_{SVC} (mmHg)**	-0.273	-0.449	-0.654	-0.312	-0.489	-0.659	-0.307	-0.489	-0.656	-0.303	-0.475	-0.650
**Q_{AoG} (L/min)**	0	0	0	0	0	0	0	0	0	0	0	0
**Q_{AD} (L/min)**	0.473	0.801	1.281	0.510	0.857	1.295	0.512	0.858	1.275	0.509	0.844	1.252
**Q_P (L/min)**	1.627	1.299	0.819	1.590	1.243	0.805	1.588	1.242	0.825	1.591	1.256	0.848
**CO/Q_S**	1	1	1	1	1	1	1	1	1	1	1	1
**Q_P/Q_S**	0.775	0.619	0.390	0.757	0.592	0.383	0.756	0.591	0.393	0.758	0.598	0.404
**$C_{O2,sa}$ (%)**	85	76	58	84	74	58	84	74	59	84	75	60

Table S5.4. CFD simulation results for the full assist mode of VEP with aortic nozzle diameter, throat diameter and atrial discharge diameter of 2.5 mm, 12 mm, and 4 mm, respectively. D_T: throat diameter, D_{AD}: atrial discharge diameter, IVC: inferior vena cava, SVC: superior vena cava, dP: pressure difference as compared to baseline condition, AoG: aortic graft, AD: atrial discharge, CO: cardiac output, Qs: systemic flow rate, $C_{o2,sa}$: systemic arterial oxygen concentration, m: atrial discharge concentration variable as described in the main text, Q_P: pulmonary flow rate.

	**Zero-DO**	**0.5-DO**	**PSC1**	**PSC2**
**P_{IVC} (mmHg)**	7.758	7.355	6.985	7.104
**P_{SVC} (mmHg)**	11.611	11.540	10.284	10.462
**dP_{IVC} (mmHg)**	-3.248	-3.470	-3.202	-3.180
**dP_{SVC} (mmHg)**	0.383	0.541	0.099	0.107
**Q_{AoG} (L/min)**	1.010	1.013	0.942	0.944
**Q_{AD} (L/min)**	0.352	0.337	0.320	0.324
**Q_P (L/min)**	2.758	2.776	2.722	2.720
**CO/Q_S**	1.481	1.482	1.449	1.450
**Q_P/Q_S**	1.313	1.322	1.296	1.295
**$C_{O2,sa}$ (%)**	92.4	92.5	92.5	92.5
**m**	0.4553	0.4502	0.4345	0.4433

Table S5.5. CFD simulation results for the occluded AoG mode of VEP with aortic nozzle diameter, throat diameter and atrial discharge diameter of 2.5 mm, 12 mm, and 4 mm, respectively. D_T: throat diameter, D_{AD}: atrial discharge diameter, IVC: inferior vena cava, SVC: superior vena cava, dP: pressure difference as compared to baseline condition, AoG: aortic graft, AD: atrial discharge, CO: cardiac output, Qs: systemic flow rate, $C_{o2,sa}$: systemic arterial oxygen concentration, m: atrial discharge concentration variable as described in the main text, Q_P: pulmonary flow rate.

	**Zero-DO**	**0.5-DO**	**PSC1**	**PSC2**
**P_{IVC} (mmHg)**	10.977	10.843	10.460	10.494
**P_{SVC} (mmHg)**	10.620	10.562	10.102	10.256
**dP_{IVC} (mmHg)**	-0.029	0.018	0.273	0.210
**dP_{SVC} (mmHg)**	-0.608	-0.437	-0.083	-0.098
**Q_{AoG} (L/min)**	0	0	0	0
**Q_{AD} (L/min)**	0.519	0.511	0.464	0.466
**Q_P (L/min)**	1.581	1.589	1.636	1.634
**CO/Q_S**	1	1	1	1
**Q_P/Q_S**	0.753	0.757	0.779	0.778
**$C_{O2,sa}$ (%)**	84.0	84.2	85.3	85.3

REFERENCES

1. Prather, R., et al., *Parametric investigation of an injection-jet self-powered Fontan circulation.* Scientific reports, 2022. **12**(1): p. 1-15.
2. Hariharan, P., et al., *Multilaboratory particle image velocimetry analysis of the FDA benchmark nozzle model to support validation of computational fluid dynamics simulations.* Journal of biomechanical engineering, 2011. **133**(4).
3. Van der Woude, S.F., et al., *The influence of respiration on blood flow in the fontan circulation: insights for imaging-based clinical evaluation of the total cavopulmonary connection.* Frontiers in Cardiovascular Medicine, 2021. **8**.
4. Hsia, T.-Y., et al., *Effects of respiration and gravity on infradiaphragmatic venous flow in normal and Fontan patients.* Circulation, 2000. **102**(suppl_3): p. Iii-148-Iii-153.
5. Dur, O., et al., *Optimization of inflow waveform phase-difference for minimized total cavopulmonary power loss.* 2010.
6. Gabbert, D.D., et al., *Heart beat but not respiration is the main driving force of the systemic venous return in the Fontan circulation.* Scientific reports, 2019. **9**(1): p. 1-10.
7. Hjortdal, V.E., et al., *Effects of exercise and respiration on blood flow in total cavopulmonary connection: a real-time magnetic resonance flow study.* Circulation, 2003. **108**(10): p. 1227-1231.
8. Hjortdal, V.E., et al., *Caval blood flow during supine exercise in normal and Fontan patients.* The Annals of Thoracic Surgery, 2008. **85**(2): p. 599-603.
